# Meta-Data Analysis to Explore the Hub of the Hub-Genes That Influence SARS-CoV-2 Infections Highlighting Their Pathogenetic Processes and Drugs Repurposing

**DOI:** 10.3390/vaccines10081248

**Published:** 2022-08-03

**Authors:** Md. Parvez Mosharaf, Md. Kaderi Kibria, Md. Bayazid Hossen, Md. Ariful Islam, Md. Selim Reza, Rashidul Alam Mahumud, Khorshed Alam, Jeff Gow, Md. Nurul Haque Mollah

**Affiliations:** 1 Bioinformatics Lab, Department of Statistics, University of Rajshahi, Rajshahi 6205, Bangladesh; parvez.mosharaf@usq.edu.au (M.P.M.); s1510124113@ru.ac.bd (M.K.K.); s1710624176@ru.ac.bd (M.B.H.); s1511124151@ru.ac.bd (M.A.I.); selim@siat.ac.cn (M.S.R.); 2 School of Business, Faculty of Business, Education, Law and Arts, University of Southern Queensland, Toowoomba, QLD 4350, Australia; khorshed.alam@usq.edu.au (K.A.); jeffrey.gow@usq.edu.au (J.G.); 3 NHMRC Clinical Trials Centre, Faculty of Medicine and Health, The University of Sydney, Camperdown, NSW 2006, Australia; rashed.mahumud@sydney.edu.au; 4 School of Accounting, Economics and Finance, University of KwaZulu Natal, Durban 4001, South Africa

**Keywords:** SARS-CoV-2 infections, selection of drug targets and agents, drug repurposing, molecular docking and dynamic simulation

## Abstract

The pandemic of SARS-CoV-2 infections is a severe threat to human life and the world economic condition. Although vaccination has reduced the outspread, but still the situation is not under control because of the instability of RNA sequence patterns of SARS-CoV-2, which requires effective drugs. Several studies have suggested that the SARS-CoV-2 infection causing hub differentially expressed genes (Hub-DEGs). However, we observed that there was not any common hub gene (Hub-DEGs) in our analyses. Therefore, it may be difficult to take a common treatment plan against SARS-CoV-2 infections globally. The goal of this study was to examine if more representative Hub-DEGs from published studies by means of hub of Hub-DEGs (hHub-DEGs) and associated potential candidate drugs. In this study, we reviewed 41 articles on transcriptomic data analysis of SARS-CoV-2 and found 370 unique hub genes or studied genes in total. Then, we selected 14 more representative Hub-DEGs (*AKT1*, *APP*, *CXCL8*, *EGFR*, *IL6*, *INS*, *JUN*, *MAPK1*, *STAT3*, *TNF*, *TP53*, *UBA52*, *UBC*, *VEGFA*) as hHub-DEGs by their protein-protein interaction analysis. Their associated biological functional processes, transcriptional, and post-transcriptional regulatory factors. Then we detected hHub-DEGs guided top-ranked nine candidate drug agents (Digoxin, Avermectin, Simeprevir, Nelfinavir Mesylate, Proscillaridin, Linifanib, Withaferin, Amuvatinib, Atazanavir) by molecular docking and cross-validation for treatment of SARS-CoV-2 infections. Therefore, the findings of this study could be useful in formulating a common treatment plan against SARS-CoV-2 infections globally.

## 1. Introduction

The epidemic of coronavirus diseases (COVID-19) has now passed the initial phase due to inconsistent RNA patterns that lead its spreading status to be out of control. Although several vaccines including BBIBP-CorV, Pfizer, BBV152, Moderna, AstraZeneca, EpiVacCorona, Sputnik, Ad5-nCoV, WIBP, and CoronaVac are now available and being dossed against SAR-CoV-2 [1,2], scientists around the world are anxious regarding their effectiveness because of the unstable pattern of RNA sequence of SARS-CoV-2 virus. For instance, recently it has been observed that fully vaccinated people are also being affected by novel SARS-CoV-2 variants around the world. In this regard, the distinct types of well-established drugs could be a supportive tonic in the treatment of supplementing vaccines against coronavirus to reduce infection rates and save lives.

However, using traditional methods, novel drug discovery is a tremendous thought-provoking, time consuming, and expensive task. The foremost challenge is to explore drug target receptors and drug candidate small molecules. Now, transcriptomics data analysis is a widely used popular approach to explore genomic biomarkers [3,4,5,6,7]. Modern high throughput technology and the gene expression data analysis techniques have revealed the potential biomarker-induced proteins which are being considered as the key drug target receptors. The repurposing of existing drugs against a disease could outweigh the de novo drug development by reducing the time and cost. Ongoing research proposes several sets of transcriptome-guided targeted genes/receptor proteins as well as different drug candidate molecules [8,9,10,11,12,13,14,15,16,17,18,19,20,21,22,23,24,25,26,27,28]. Nonetheless, the comparative discussion regarding the consistency and inconsistency of the drug target molecules (i.e., Hub-DEGs) among the distinct articles of COVID-19 has not been presented yet. In our literature review, we also noticed that there was not any set of genes/receptor proteins and/or drug molecules regarding COVID-19 infection that are commonly reported across studies. It may happen due to study design, data collection methods, and the environment.

**Figure 1:**
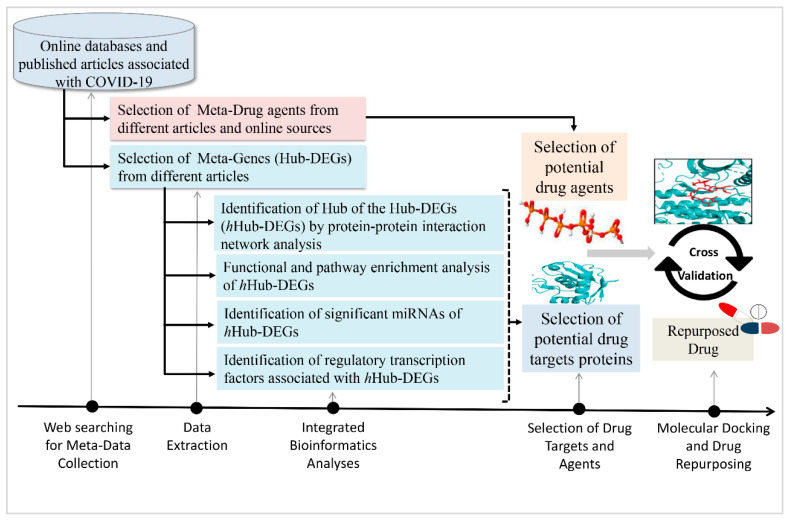
The road map of this research.

Therefore, it may be difficult to develop a common therapeutic treatment plan against SARS-CoV-2 infection. To make a comparatively more common therapeutic development strategy for all, it is required to find more representative Hub-DEGs set which will be the global key drug target molecules for the human body. There are several research groups working with COVID-19 research [8,9,10,11,12,13,14,15,16,17,18,19,20,21,22,23,24,25,26,27,28], but so far this issue has not been reported yet. The goal of this study was to provide more representative Hub-DEGs from all published studies by means of hub of Hub-DEGs (hHub-DEGs) and the associated potential drug candidates. The pipeline of this study is given in Figure 1.

## 2. Materials and Methods

In this project, the necessary meta-genes and re-purposable meta-drug agents were collected from different online sources and published articles. Then, the integrated bioinformatics analysis approach was utilized to investigate the most effective drug target biomolecules and potential drug agents for SARS-CoV-2.

### 2.1. Metadata Sources and Descriptions

In this study, we collected metadata for both drug targets and drug agents that were associated with SARS-CoV-2 as described below:

#### 2.1.1. Collection of Hub-DEGs to Explore Drug Targets

Several research groups have already published different sets of Hub-DEGs that are associated with SARS-CoV-2 infections. Here, Hub-DEGs indicates the key differentially expressed genes between SARS-CoV-2 infections and control samples. We collected 41 published articles that suggested SARS-CoV-2-causing Hub-DEGs through the Google Scholar search engine. We found in total 370 unique Hub-DEGs/studied genes (Table 1) by reviewing those 41 articles.

#### 2.1.2. Collection of Drug Agents

Among the 3410 FDA-approved antiviral drugs, the top listed SARS-CoV-2 3CL protease-guided 88 drugs that were collected from Beck et al., 2020 [52] were considered as drug Set A. We have also collected a total of 87 published drugs from our reviewed articles (Table 1) and consider them as Set B. Then, we have taken common and uncommon drugs from Set A and B, (*AUB*) = 145 drugs (Supplementary File S1). These drugs would be evaluated against the potential drug targets by molecular docking simulation study.

### 2.2. Methods

The integrated bioinformatics and system biology analysis approaches were utilized as described below. This widely used analytical approach has been utilized in transcriptome guided network-based analysis to identify leading biomolecules [50,53,54].

#### 2.2.1. Protein–Protein Interaction (PPI) Network Analysis of Hub-DEGs

The PPI network analyses of DEGs are now widely being used to explore key proteins. The PPI network that is based on all sets of Hub-DEGs encoded proteins was constructed using the STRING database [55] to detect hHub-DEGs. The NetworkAnalyst and Cytoscape 3.7.2 (Boston, MA, USA) [53] were utilized to perform topological analyses of PPI network and visualization.

#### 2.2.2. Functional and Pathway Enrichment Analysis of hHub-DEGs

Functional enrichment, annotation, and over-representation analysis [54,56,57,58], namely, biological processes (BP), molecular functions (MF), and cellular components (CC) and the Kyoto Encyclopedia of Genes and Genomes (KEGG) pathways were retrieved from the hHub-DEGs. The functional and pathway enrichment analysis was performed by using g:GOSt software that was implanted in the g:Profiler web server to reveal the significant GO terms of BP, MF, CC, and KEGG pathways of hHub-DEGs for SARS-CoV-2 infections. Statistical significance was defined by the adjusted *p*-values < 0.05 and the Benjamini and Hochberg [59] procedure was considered for FDR controlling.

#### 2.2.3. Regulatory Network Analysis of hHub-DEGs

To explore Hub-TFs of hHub-DEGs, we constructed the interaction network between TFs and hHub-DEGs based on the JASPAR database [60]. Similarly, the miRNA and hHub-DEGs interaction network was constructed based on the TarBase V8.0 [61] database. These key regulatory biomolecules were selected based on the highest topological matrices (degree of connectivity and betweenness) that were applied on the interaction network by using the NetworkAnalyst tool [53].

#### 2.2.4. Association of hHub-DEGs with Comorbidities

The prognostic performance of hHub-DEGs based on lung cancer patient data was observed by SurvExpress [62], an online biomarker validation tool. The SurvExpress utilizes the log rank statistic to test the significance.

#### 2.2.5. Drug Repurposing by Molecular Docking Simulation

Basically, the molecular docking simulation interprets the potential drug components based on computational binding affinity with the drug target molecules. In this study, the docking analysis was performed among the drug target key receptors biomolecule (i.e., hHub-DEGs, TFs) and 145 drug agents or small compounds (Supplementary File S1). The molecular docking simulation requires 3-Dimensional (3D) structures of both receptor proteins and candidate drugs.

The 3D structures of receptors were downloaded from the Protein Data Bank (PDB) [63] and SWISS-MODEL [64]. The PubChem database [65] was used to retrieve the 3D structures of 145 meta-drug agents. The “Discovery Studio Visualizer” was used to visualize the 3D structures of the protein interfaces [66]. The receptor proteins were preprocessed by removing solvent molecules (water) and all hetero atoms, and adding charges using AutoDock tools and also a grid box was generated over the entire surface of the receptors [67]. The drug agents were minimized energy through the Avogadro using the mmff94 force field along with the steepest descent optimization algorithm and a total number of 200 minimization steps [68] and pre-processed by AutoDock tools which convert each ligand into an acceptable format PDBQT [67], respectively. Subsequently, molecular docking between the receptors and drug agents were performed to calculate their binding affinities (kcal/mol) by using AutoDock Vina [69]. The receptor-ligand complexes with binding affinities that were less that −7.0 (kcal/mol) were considered as the significant complexes. The number of iterations per pocket was used with the default value of 8. The PyMol [70] was used to analyze the docked complexes for surface complexes, types, and distances of non-covalent bonds.

Then, we validated our proposed drug agents by a molecular docking study with the top listed 11 previously published receptor proteins (CXCL8, IL6, TNF, TP53, IL1B, MMP9, NFKBIA, PTGS2, ICAM1, STAT1, CCL2) for novel SARS-CoV-2 infections (Table 1). Each of these 11 receptors was detected as a hub-gene/protein in at least 5 articles. There was no other independent receptor that is published in more than 5 articles.

## 3. Results

### 3.1. Basic Characteristics of the Selected Studies

In this meta-data analysis, we have collected mainly the hub-DEGs information of the published articles that were related to the COVID-19. To identify the dysregulated genes, most of the articles analyzed the RNA-Seq and/or microarray transcriptomics data of COVID-19 from Gene Expression Omnibus (GEO) and some were collected from various published sources (i.e., different databases, other published sources). One article used the SNP data to identify the significant target genes for COVID-19. The LIMMA, DESeq2, classical *t*-test, and EdgeR were the most used statistical tests to identify the DEGs from the gene expression datasets. Additionally, some articles analyzed other datasets of different comorbidities and compared the findings with COVID-19 (Supplementary Table S1). The drug information for COVID-19 treatments were also reported in some published articles. We have found 531 hub-DEGs in total (i.e., 370 unique genes) from the reviewed articles in this study that were used in PPI network.

### 3.2. Identification of Hub of Hub-Proteins (hHub-Proteins)

**Figure 2:**
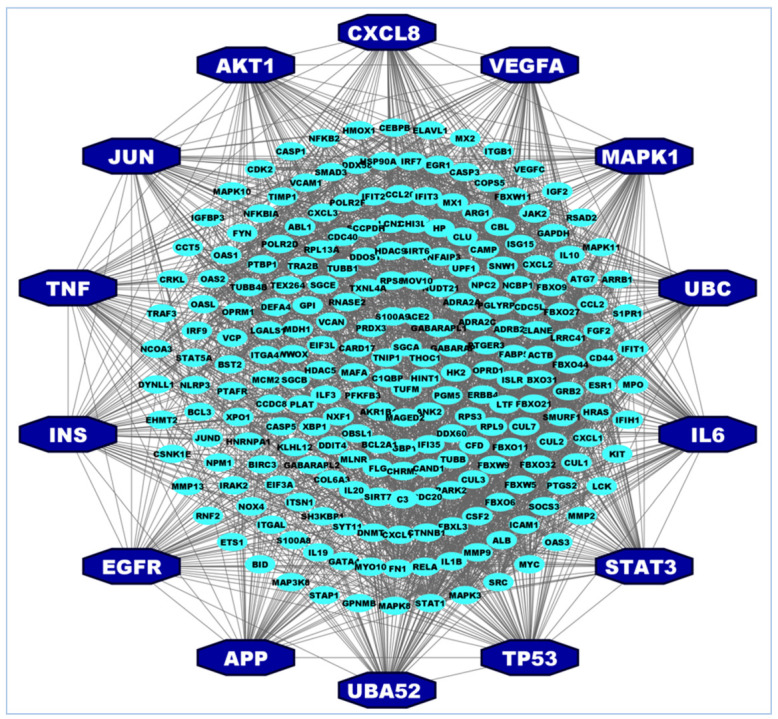
Protein–protein interaction (PPI) network analysis of Hub-DEGs-detected hHub-DEGs. The nodes in octagon shape with a blue color indicate the hHub-DEGs and a small ellipse indicates hub DEGs.

To identify the hHub-proteins, the PPI network analysis was utilized by using all collected the hub-DEGs from the selected articles. The PPI network of the hub-DEGs revealed the key hHub-proteins that were chosen based on the degree of connectivity, closeness, and betweenness of the nodes in the network. The top 14 hHub-proteins are AKT1, APP, CXCL8, EGFR, IL6, INS, JUN, MAPK1, STAT3, TNF, TP53, UBA52, UBC, and VEGFA (Figure 2) that were found by PPI network. These top hHub-proteins would be focused for the pre-clinical potential drug target molecule that may open a new era of therapeutic targets.

### 3.3. Functional and Pathway Enrichment Analysis of hHub-DEGs

The GO functional enrichment analysis revealed that our proposed hHub-DEGs were significantly enriched with abundant number of biological processes (BPs), molecular functions (MFs), and cellular components (CCs) (Table 2). Table 2 shows top 10 significantly enriched GO-terms for each of three categories (MFs, BPs, and CCs). These functions and pathways are highly connected with the COVID-19-related biological functional pathways in the host which are crucial for developing therapeutic targets. The major GO molecular functions (MF) are namely, enzyme binding, identical protein binding, phosphatase binding, cytokine receptor binding; the major GO biological process (BP) showed that the positive regulation of cellular biosynthetic process, regulation of DNA-binding transcription factor activity, positive regulation of protein phosphorylation, positive regulation of protein modification process, response to endogenous stimulus, and cellular response to organic substance. Moreover, the intracellular organelle lumen, membrane-enclosed lumen, membrane endosome, endoplasmic reticulum, and cell periphery are the significant cellular areas for the hHub-DEGs showed in the functional enrichment analysis (Table 2).

**Figure 3:**
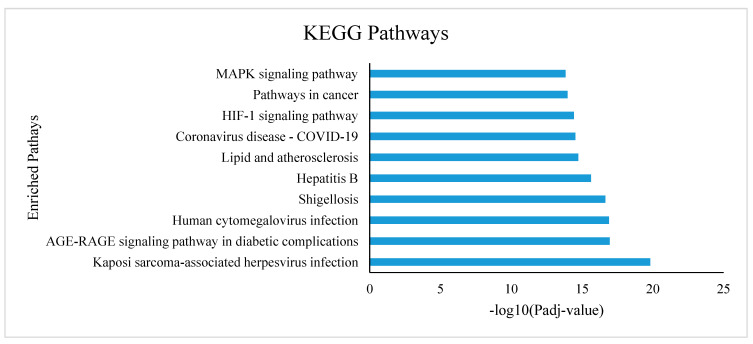
The KEGG pathways enriched by the proposed hHub-DEGs.

On the other hand, the hHub-DEGs shared significant KEGG pathways, noticeably Kaposi sarcoma-associated herpesvirus infection, AGE-RAGE signaling pathway in diabetic complications, Human cytomegalovirus infection, Shigellosis, Hepatitis B, Lipid and atherosclerosis, Coronavirus disease-COVID-19, HIF-1 signaling pathway, and pathways in cancer (Figure 3). Among the top significant KEGG pathways, “the Human cytomegalovirus infection”, “Shigellosis”, and “Coronavirus disease-COVID-19” (Figure 3) are the most important pathways which are crucial for mortality due to COVID-19 infection.

### 3.4. Transcriptional and Post-Transcriptional Regulatory Factors

**Figure 4:**
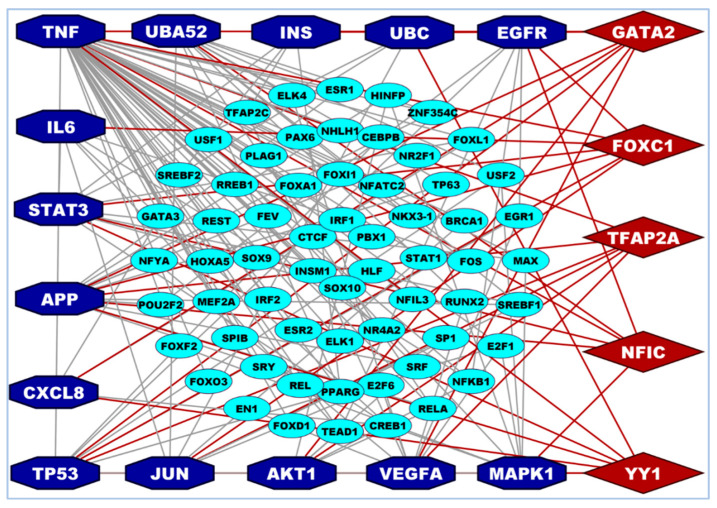
TFs versus hHub-DEGs interaction network that was detected the key regulatory TFs of hHub-DEGs. Here, hHub-DEGs were marked as a blue color with octagon shape in both A and B. The key TFs proteins were marked as a red color with a hexagonal shape and small ellipses represents other TFs.

**Figure 5:**
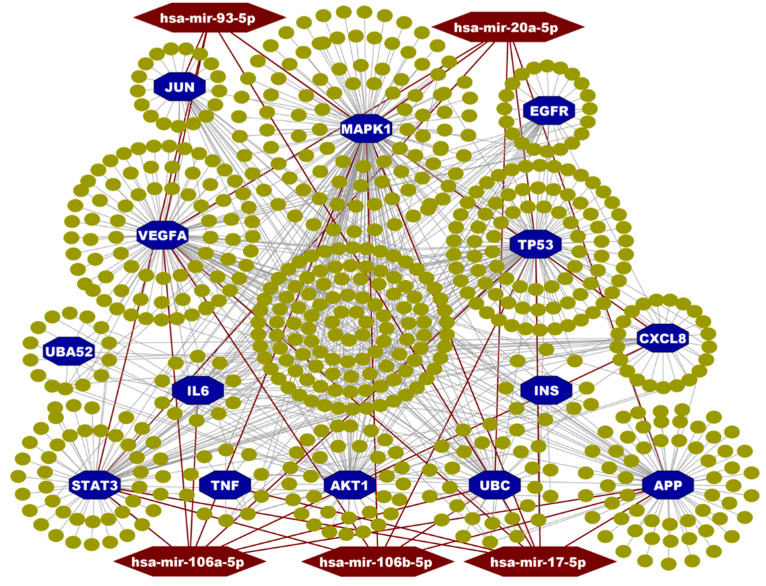
The miRNAs versus hHub-DEGs interaction network that were detected the key regulatory miRNAs of hHub-DEGs. Here, hHub-DEGs were marked as a blue color with octagon shape in both A and B. The key miRNAs were marked as a red color with a hexagonal shape and small ellipses represents other miRNAs.

The interaction network of regulatory TFs-target hHub-DEGs and the miRNA- hHub-DEGs show the substantial TFs and the miRNAs that regulate the hHub-DEGs. The transcription factors GATA2, FOXC1, TFAP2A, NFIC, and YY1 (Figure 4) and the miRNAs namely, miR-106a-5p, miR-17-5p, miR-20a-5p, miR-106b-5p, and miR-93-5p (Figure 5) were found from the network as key signaling and regulatory factors that were associated with SARS-CoV-2 infection in humans which play the key regulatory roles for hHub-DEGs (Figure 5).

### 3.5. Drug Repurposing by Molecular Docking

We selected 14 hub receptor proteins corresponding to our proposed 14 hHub-DEGs and 5 TFs proteins corresponding to the key regulators of those hHub-DEGs, as the *m* = 19 drug targets and 145 drug agents (Supplementary File S1). We downloaded the 3D structure of 15 drug target proteins (MAPK1, EGFR, CXCL8, STAT3, UBC7, IL6, AKT1, GATA2, TNF, YY1, UBA52, VEGFA, JUN, INS, and APP) from PDB [63] with source codes 6g54, 3ika, 4xdx, 6njs, 3h8k, 1il6, 2uzr, 5o9b, 1tnf, 1ubd, 2hth, 2vpf, 1jun, 6ver, and 4pwq, respectively, as well as the 3D structure of remaining four drug target TFs protein (TP53, FOXC1, NFIC, and TFAP2A) that were collected from SWISS-MODEL [64] using UniProt [71] ID of P04637, Q12948, P08651, and P05549, respectively. The 3D structures of 145 drugs were downloaded from the PubChem database [65] as mentioned previously.

**Figure 6:**
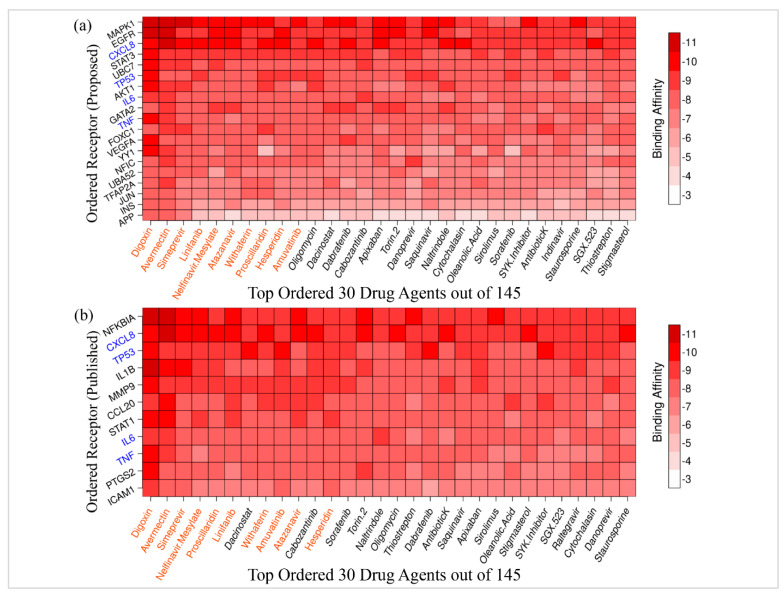
Binding affinity scores that were calculated by Autodock-vina. The color bar indicates the score levels, where deeper and lighter reds indicated the strong and weak binding, respectively. (**a**) The binding affinity scores based on the top-ranked 30 drug agents out of 145 on the *X*-axis and ordered 19 target proteins (proposed) on the *Y*-axis (Supplementary File S2). (**b**) The binding affinity scores that were based on the top-ranked 30 drug agents out of 145 on the *X*-axis and top-ranked 11 target proteins (previously published) on the *Y*-axis; see details in (Supplementary File S3). The receptors with blue color indicate the common receptors between the proposed and previously published top-ranked receptors.

Molecular docking analysis between our proposed receptors and meta-drug agents were performed to calculate the binding affinity scores. Then, for the selection of drug agents as candidate drugs, we ordered the target proteins according to the row sums of the binding affinity matrix A = (Aij) and the column sums of the drug agents. Figure 6a shows the image of the docking score matrix $A=\left(A_{\mathrm{ij}}\right)\;$ corresponding to the ordered target proteins on the *Y*-axis and *n* = 30 top ranked drug agents on the *X*-axis. We observed that first two top lead compounds (lead1: Digoxin and lead2: Avermectin) produced binding affinities that were negatively larger than −8.0 kcal/mol with 15 receptor proteins out of 19. The next (3–10)th top lead compounds (lead3: Simeprevir, lead4: Linifanib, lead5: Nelfinavir Mesylate, lead6: Atazanavir, lead7: Withaferin, lead8: Proscillaridin, lead9:, Hesperidin and lead10: Amuvatinib) produced binding affinities that were negatively larger than −7.0 kcal/mol with our proposed 12 receptor proteins out of 19. The remaining 20 lead compounds also produced good binding affinities with top ordered proposed 10 receptor proteins (Figure 6a). Therefore, we considered the top 10 lead compounds (Digoxin, Avermectin, Simeprevir, Linifanib, Nelfinavir Mesylate, Atazanavir, Withaferin, Proscillaridin, Hesperidin, and Amuvatinib) as the most possible candidate drug agents for SARS-CoV-2 infections and the 2D structures of these drug agents were provided in the Supplementary Table S2a as well as their pairwise ligand similarities scores in Supplementary Table S2b, were calculated by using the Tanimoto Coefficients (TC) [72,73].

To investigate the binding performance of the proposed candidate drugs against the state-of-the-art alternative independent receptors, we considered SARS-CoV-2 infection causing top-ranked 11 hub-DEGs (CXCL8, IL6, TNF, TP53, IL1B, MMP9, NFKBIA, PTGS2, ICAM1, STAT1, CCL2) that were previously published in at least five articles (Table 1). The 3D structures of these 11 proteins were collected from the Protein Data Bank (PDB) with codes 2vpf, 1il6, 1tnf, 4mzi, 9ilb, 1gkc, 1nfi, 1cx2, 1p53, 1bf5, and 3ifd, respectively. Then, molecular docking interactions of the top drug agents with published 11 KGs were performed. Their binding affinities (kcal/mol) were visualized in Figure 6b. Then we ordered the publicly available receptor proteins in descending order of row sums of the binding affinity matrix A = (*A_ij_*) and drug agents according to the column sums, in the same manner as before. Then, we observed that first ten top compounds that produced binding affinities that were negatively larger than −7.0 kcal/mol with all published independent receptor proteins. Then, we selected common nine lead compounds (Digoxin, Avermectin, Simeprevir, Nelfinavir Mesylate, Proscillaridin, Linifanib, Withaferin, Amuvatinib, Atazanavir) as the candidate drug agents from the top-ranked 10 lead compounds as displayed in Figure 6a,b for SARS-CoV-2 infections.

For example, Table 3 shows the summary results of the interacting properties of two potential target proteins (*MAPK1* and *EGFR*) with the top three lead compounds (Digoxin, Avermectin, Simeprevir) that achieved the highest binding affinity scores. The interacting complex (3D), schematic diagram (2D), and the interaction neighbor residues (with 4 Å radius of drug) are represented in Table 3. The complex MAPK1-Digoxin was formed with two conventional hydrogen bonds at ARG191 and TRP192, five alkyl bonds at ALA52, LEU170, VAL39, LUE156, and ILE84. The complex between EGFR and Avermectin was formed with one conventional hydrogen bond at VAL876; one carbon hydrogen bond at ASP855; five alkyl bonds at VAL726, LYS745, ILE878, LYS879, and ARG858; and only one pi-alkyl bond at PHE723. The complex MAPK1-Simeprevir was formed two conventional hydrogen bonds at LYS151 and SER153; two pi-sigma bonds at ILE31 and LEU156; and five alkyl bonds at ALA52, VAL39, LYS54, LEU107, and LEU156. The docking poses of the proposed 10 ligands with the proposed top ordered six targets (MAPK1, EGFR, CXCL8, STAT3, UBC7, TP53) were displayed in Supplementary Figure S2. We observed that our suggested top-ranked 10 ligands significantly bind to the same pockets of the top-ranked two targets MAPK1 and EGFR (Supplementary Figure S1a,b). Few ligands bind to the different pockets for the (3–6)th-ranked targets (UBC7, CXCL8, STAT3, TP53) (Supplementary Figure S1c–f). Similarly, we also observed that previously published the top-ranked four receptors (uncommon) NFKBIA, CXCL8, MX1, and IRF7 are significantly targeted by our suggested ligands (Supplementary Figure S2a–d).

## 4. Discussion

Among the selected 39 published studies, it was commonly suggested the drug target proteins, called hub-proteins, encoded from the hub-DEGs for COVID-19. We have observed that the reported hub-DEGs were not consistent from the COVID-19 perspective. In total, 370 unique hub-DEGs were found from 41 articles in this study. The reported hub-DEGs were not consistent among the articles due to the different conditions of data collection (Table 1). The crucial key biomarkers play the vital role of biological functions in all the living organs. The deeper understanding of the molecular mechanism of these potential biomarkers is essential for all diseases. The recent transcriptional data analysis-based research is being utilized to focus on the pre-clinical prominent drug candidate biomarkers [4,5,6,7]. Due to reporting inconsistency among the published drug target hub-DEGs of COVID-19 host cell, the current study focused on identifying global key hub-DEGs (hHub-DEGs) using the metadata of published articles through a multi-omics data integration framework (Figure 1). The prominent substantial hHub-proteins and their associated regulatory TFs and miRNAs were identified which are related to SARS-CoV-2 infection and proliferation. The metadata analysis identified 14 key hHub-DEGs, 5 TFs, and 5 miRNAs as the top regulatory factors using an integrated bioinformatics framework analysis.

The hHub-DEGs were identified using the PPI network analysis of the selected published 370 unique hub-DEGs which revealed 14 key hHub-DEGs namely *AKT1*, *APP*, *CXCL8*, *EGFR*, *IL6*, *INS*, *JUN*, *MAPK1*, *STAT3*, *TNF*, *TP53*, *UBA52*, *UBC,* and *VEGFA*. The *AKT1* gene encoded protein-kinase supports cell growth and proliferation, cell differentiation, and cell survival. This protein also helps normal development and function of the nervous system and also has the potential to cause normal cells to become cancerous when it mutates [74,75,76,77]. Recent studies show that the *AKT1* gene is one potential drug target for COVID-19 treatment and also has a greater involvement in SARS-CoV-2 viral infection related complexity [78,79,80]. Amyloid Protein Precursor (*APP*) gene duplication is associated with Cerebral Amyloid Angiopathy which leads to intracerebral hemorrhagic stroke. This triggers the critical condition for COVID-19-infected persons that have Alzheimer’s, Down syndrome, and cardiovascular disease [81,82]. *CXCL8* (also known as IL-8) is a major inflammatory mediator and it’s receptors are highly connected with the development of various colorectal cancer and its liver metastases [83] as well as it enhances the major human neutrophils conscription [84]. The cell growth process and lung cancer development is influenced by the *EGFR* gene [85]. Studies have suggested that the EGFR protein incorporates the novel coronaviruses by making bonds with S protein which facilitates the endocytosis process into the host cell [86,87] by SARS-CoV-2. The EGFR protein also triggers the MAPK pathways after binding with the spike protein of SARS-CoV-2 virus which is also important for viral entry [88]. The MAPK family proteins are also associated with the cytokine signaling after COVID-19 infection [89,90]. The *MAPK1* gene is related with the regulation of immunity and inflammation [15] when the *TNF* is linked with the immune regulatory, pro-inflammatory [70,91], and anti-viral functions [92]. One of the most important hHub-DEGs, *IL6,* is related to the interleukin (IL) regulatory pathway genes which are critical for the significant pathophysiological functional mechanisms, namely systemic inflammation and cytokine release syndrome [91,93,94] which plays the major role in the cytokine storm [95,96]. The *IL-6* can also be used as an early warning diagnosis and treatment index of COVID-19 [97,98] and also treated as the molecule for assessing the severity of infection and judge prognosis [99,100] and the response to treatment [101,102]. The c-Jun type gene, namely *JUN* is one of the important host proteins involving in HCoV infectious bronchitis virus [28,103]. The *TP53* and *UBC* hHub-DEGs are involved in cell deaths that occurs from protein misfolding and aggregation [38]. The IFN-γ-mediated signaling function, apoptosis, and proteasomal degradation of CD4^+^ T-cells are mediated by the vital proteins namely, *TP53*, and *CASP3*, *UBA52*, and *UBC*, respectively [104]. The study revealed that the cancer growth is suppressed and the radio sensitivity is amplified by the activities of ubiquitin C (*UBC*) in NSCLC cells [105] The above discussions and the importance of the hHub-DEGs clearly demonstrated that these are the most important proteins for COVID-19 infection, diagnosis, and proliferation which shows them as significant drug target proteins for COVID-19 treatment.

The functional annotation and enrichment analysis of the hHub-DEGs showed significant enrichment in various biological, molecular, and cellular functions into the host cells. Most of the hHub-DEGs showed involvement in different functional pathways. The top significant molecular functions demonstrated that the hHub-DEGs are associated with different binding activities which included proteins, enzymes, phosphatase, and signaling receptors (Table 2). The binding activities indicates the regulatory and functionality of the hHub-DEGs. Most importantly, the hHub-DEGs enriched the cytokine receptor binding and ubiquitin protein ligase binding molecular functions (MF) which are signifying the COVID-19-induced cytokine storm in the affected patients. The top Gene Ontology (GO) terms of biological processes (BP) are suggesting that the positive regulation of cellular biosynthetic process, regulation of DNA-binding transcription factor activity, and the protein phosphorylation-associated regulation and metabolic functions are the most significant (Table 2). The other top significant BPs, namely the positive regulation of protein modification process, response to endogenous stimulus, and the cellular response to organic substance is also enriched by our identified hHub-DEGs. The cellular presence of the proposed hHub-DEGs reveal that the cellular lumen, membrane, endosome, endoplasmic reticulum, cell periphery, extracellular areas, are significantly enriched.

The KEGG pathway enrichment analysis of the hHub-DEGs represents the top significant pathways that are shared by the hHub-DEGs are namely, Kaposi sarcoma-associated herpesvirus infection, AGE-RAGE signaling pathway in diabetic complications, Human cytomegalovirus infection, Shigellosis, Hepatitis B, Lipid and atherosclerosis, Coronavirus disease-COVID-19, HIF-1 signaling pathway, and pathways in cancer (Figure 3). Most of the hHub-DEGs are involved in the top 10 significant KEGG pathways. Among the enriched significant pathways, the Coronavirus disease-COVID-19-related pathways is the most significant pathway which involved most of the hHub-DEGs notably, *CXCL8*, *EGFR*, *IL6*, *JUN*, *MAPK1*, *STAT3*, *TNF,* and *UBA52*. The functions and pathway enrichment analyses are representing the significance of the hHub-DEGs in that they are very crucial to COVID-19 infection, diagnosis, and treatment.

The TFs vs. hHub-DEGs interaction network revealed that the GATA2, FOXC1, TFAP2A, NFIC, and YY1 are prominent and important TFs that are associated with the hHub-DEGs (Figure 5). Among the TFs, the Alzheimer’s disease, basal-like breast cancer (BLBC), and tissue invasion are highly related with FOXC1 TF [106,107]. The GATA2 TF is related to kidney and breast cancer-related pathway when the higher expression pattern of YY1 TFs increases the tumour size and higher TNM stage [108,109,110]. The contribution of TFAP2A was found for the malignant progression of lung cancer [111]. The NFIC TFs have greater involvement with the tumor genesis of breast cancer, gastric cancer, and glioma [112,113,114]. Also, the identified TFs have a significant involvement in various biological functions and pathways [4,5,6,7,115]. The interaction network among the miRNAs and hHub-DEGs fetched the five key miRNAs (miR-106a-5p, miR-17-5p, miR-20a-5p, miR-106b-5p and miR-93-5p) that were linked with the hHub-DEGs-encoded proteins. The miR-106a-5p miRNA is highly interconnected with the CD4^+^ T-cells regulation and works as a tumor suppressor by regulating the *VEGFA* gene [116,117]. The miR-106b-5p and miR-17-5p miRNAs are related to different cancers and bone formation pathways (i.e., cervical and breast cancer) when the miR-20a-5p, miR-106b-5p, and miR-93-5p are related to cervical cancer [118,119,120,121]. The survival analysis showed that the prognostic power of the hHub-DEGs clearly discriminate between the low and high-risk groups (Supplementary Figure S3). The survival curve was based on lung cancer data which indicates that the patients with lung-related comorbidities have a greater risk to be infected and higher mortality rate compared to others.

Based on the molecular docking binding scores, the top ranked nine drugs (Digoxin, Avermectin, Simeprevir, Nelfinavir Mesylate, Proscillaridin, Linifanib, Withaferin, Amuvatinib, Atazanavir) were selected as potential drug candidates for the preventing of SARS-CoV-2 infection, where the first three drugs (Digoxin, Avermectin/Ivermectin, Simeprevir) showed the strongest binding affinities with maximum target proteins (Figure 6a,b). Studies suggested that COVID-19-affected patients had a greater risk of occurrence of congestive heart failure (CHF), atrial fibrillation or flutter, and certain cardiac arrhythmias which increases the morbidity and mortality significantly [122,123]. In these cases, the Digoxin has been injected to reduce the heart disease risk, entrance of coronavirus into the cells, and the suppression of a cytokine storm [124], although it has toxicity which causes the adverse events for instance vomiting, abdominal pain, dizziness, and delirium [122,125]. In spite of a lack of deeper knowledge about the activities of Avermectin/Ivermectin against SARS-CoV-2 virus, it has been practiced to treat COVID-19-affected patients [126] during the pandemic situation of the last couple of years. Ivermectin has been widely used for the treatment of virus infection [127,128] and many researchers have suggests that it has potential therapeutic power for COVID-19 also [129,130]. On the other hand, Simeprevir, a protease inhibitor has been used for hepatitis C virus (HCV) infection treatment [131,132]. The literature supports that Simeprevir inhibits SARS-CoV-2 viral replication by targeting the important viral polyproteins and cleaves them in vitro condition [133,134]. On the other hand, molecular docking analysis for the two top ranked proteins, MAPK1 and EGFR, showed maximum binding affinity with the top ranked drug components (Figure 6). The MAPK1 and the EGFR are very important proteins that are associated with the COVID-19 infection as discussed above. Therefore, the most importantly proposed three drug candidates might play a significant role for therapeutic development against SARS-CoV-2 variants. Moreover, further diverse research and experimental wet lab validations are being emphasized to investigate the effectiveness of the proposed target proteins and the drugs in clinical trials.

## 5. Conclusions

The global crisis due to the COVID-19 pandemic has caused millions of deaths and great economic losses. The analyses revealed the drug target proteins in host cells which were not conserved across the studies. Therefore, the current study focused on accumulating the published information about the drug target proteins to decode the more comprehensive drug target molecules in host human cells using an integrated bioinformatics approach. We identified 14 hHubGs (*AKT1*, *APP*, *CXCL8*, *EGFR*, *IL6*, *INS*, *JUN*, *MAPK1*, *STAT3*, *TNF*, *TP53*, *UBA52*, *UBC*, *VEGFA*) that were related to COVID-19 in host cells along with 5 TFs (GATA2, FOXC1, TFAP2A, and YY1) as the transcriptional and 5 miRNAs (miR-106a-5p, miR-17-5p, miR-20a-5p, miR-106b-5p, and miR-93-5p) as the post-transcriptional regulators. The GO functional and KEGG pathway enrichment analysis showed that the hHub-DEGs are significantly associated with infections. The molecular docking simulation analysis showed the significant drugs based on their binding affinities with the drug target hHub-DEGs. Finally, hHub-DEGs guided the top ranked nine drugs (Digoxin, Avermectin, Simeprevir, Nelfinavir Mesylate, Proscillaridin, Linifanib, Withaferin, Amuvatinib, Atazanavir) which were identified by molecular docking simulation and cross-validation. These drugs need more attention for their experimental and clinical validation to be used as therapeutics for COVID-19 treatment.

## Figures and Tables

**Table 1:** Hub genes/proteins that were associated with SARS-CoV-2 infections in different studies.

SL	Articles & Datasets	Hub-Genes/Proteins	Number of Proteins
1	Caradonna, A et al., 2022 [29]	ACE2, APP	2
2	Hanming Gu et al., 2020 [30]	NFKBIA, C3, CCL20, BCL2A1, BID	5
3	Kang Soon Nan et al., 2021 [18]	ALB, CXCL8, FGF2, IL6, INS, MMP2, MMP9, PTGS2, STAT3, VEGFA	10
4	Hanming Gu et al., 2020 [31]	CDC20, NCBP1, POLR2D, DYNLL1, FBXW5, LRRC41, FBXO21, FBXW9, FBXO44, FBXO6	10
5	Rahila Sardar et al., 2020 [19]	HMOX1, DNMT1, PLAT, GDF1, ITGB1	5
6	Hanming Gu et al., 2020 [21]	FLOC, DYNLL1, FBXL3, FBXW11, FBXO27, FBXO44, FBXO32, FBXO31, FBXO9, CUL2	10
7	Tian-Ao Xie et al., 2020 [8]	CXCL1, CXCL2, TNF, NFKBIA, CSF2, TNFAIP3, IL6, CXCL3, CCL20, ICAM1	10
8	Jung Hun Oh et al., 2020 [9]	GATA4, ID2, MAFA, NOX4, PTBP1, SMAD3, TUBB1, WWOX	8
9	Basavaraj Vastrad et al., 2020 [20]	TP53, HRAS, CTNNB1, FYN, ABL1, STAT3, STAT1, JAK2, C1QBP, XBP1, BST2, CD99, IFI35, MAPK11, RELA, LCK, KIT, EGR1, IL20, ILF3, CASP3, IL19, ATG7, GPI, S1PR1	25
10	Kartikay Prasad et al., 2020 [22]	STAT1, IRF7, IFIH1, MX1, ISG15, IFIT3, OAS2, DDX58, IRF9, IFIT1, OAS1, OAS3, DDX60, OASL, IFIT2	15
11	Gurudeeban Selvaraj et al., 2021 [32]	MYC, HDAC9, NCOA3, CEBPB, VEGFA, BCL3, SMAD3, SMURF1, KLHL12, CBL, ERBB4, CRKL	12
12	Md. Shahriare Satu et al., 2021 [24]	MARCO, VCAN, ACTB, LGALS1, HMOX1, TIMP1, OAS2, GAPDH, MSH3, FN1, NPC2, JUND, CHI3L1, GPNMB, SYTL2, CASP1, S100A8, MYO10, IGFBP3, APCDD1, COL6A3, FABP5, PRDX3, CLEC1B, DDIT4, CXCL10, CXCL8	27
13	Tasnimul Alam Taz et al., 2020 [25]	VEGFA, AKT1, MMP9, ICAM1, CD44	5
14	Mohammad Ali Moni et al., 2020 [26]	MX1, IRF7, BST2	3
15	Tania Islam et al., 2020 [27]	BIRC3, ICAM1, IRAK2, MAP3K8, S100A8, SOCS3, STAT5A, TNF, TNFAIP3, TNIP1	10
16	Yadi Zhou et al., 2020 [28]	JUN, XPO1, NPM1, HNRNPA1	4
17	Ge C et al., 2020 [10]	MMP13, NLRP3, GBP1, ADORA2A, PTAFR, TNF, MLNR, IL1B, NFKBIA, ADRB2, IL6	11
18	Aishwarya et al., 2020 [11]	IGF2, HINT1, MAPK10, SGCE, HDAC5, SGCA, SGCB, CFD, ITSN1, EHMT2, CLU, ISLR, PGM5, ANK2, HDAC9, SYT11, MDH1, SCCPDH, SIRT6, DTNA, FN1, ARRB1, MAGED2, TEX264, VEGFC, HK2, TXNL4A, SLC16A3, NUDT21, TRA2B, HNRNPA1, CDC40, THOC1, PFKFB3	34
19	Saxena, A. et al., 2020 [12]	STAP1, CASP5, FDCSP, CARD17, ST20, AKR1B10, CLC, KCNJ2-AS1, RNASE2, FLG	10
20	Tao Q et al., 2020 [13]	MAPK3, MAPK1, MAPK8, IL10, TNF, CXCL8, IL6, PTGS2, TP53, CCL2, CASP3, IL1B	12
21	Zhang N et al., 2020 [14]	CXCL10, ISG15, DDX58, MX2, OASL, STAT1, RSAD2, MX1, IRF7, OAS1	10
22	Han L et al., 2020 [15]	IL6, TNF, IL10, MAPK8, MAPK3, CXCL8, CASP3, PTGS2, TP53, MAPK1	10
23	Tian J et al., 2020 [33]	CXCL8, CXCL2, CXCL10, ADRA2A, ADRA2C, CHRM2, PTGER3, OPRM1, OPRD1, JUN.	10
24	Jha PK et al., 2021 [34]	SMAD3, STAT1, SH3KBP1, HDGF, TUBB, NFKB2, ETS1, UBC, TUFM, TRAF3, CCT5, RPL9, TUBB4B, CSNK1E, S100A9	15
25	Ramesh P et al., 2020 [35]	ELANE, MPO, ARG1, DEFA4,CAMP, MMP9, LTF, LCN2,PGLYRP1,HP	10
26	Li Zhonglin et al., 2020 [36]	DDOST, UPF1, HIST2H2A, ITGAL, EGFR, CXCL1, DYNLL1, POLR2F, RPL13A, FBXO11, CSNK1E	11
27	Li G et al., 2020 [37]	RPS3, RPS8, PRS9, VCP, LARP1, UBA52, PRKN, EIF3A, EIF3L, SRC, CASP1, RIPK, ACE2	13
28	Prasad K et al., 2021 [38]	MOV10, NXF1, APP, ELAVL1, CUL3, XPO, TP53, EGFR, MCM2, MYC, COPS5, ESR1, UBC, FN1, CUL7, VCAM1, RNF2, CUL1, SIRT7, CAND1, OBSL1, HSP90AA1, CDK2, NPM1, GRB2, FBXO6, CDC5L, GABARAPL2, VCP, CCDC8, GABARAPL1, CUL2, SNW1, ITGA4, GABARAP	35
29	Fangzhou Liu et al., 2021 [39]	AKT1, TP53, TNF, IL6, BCL2L1, ATM	6
30	Zulkar Nain et al., 2020 [40]	NFKBIA, BUB3, EIF2S3, GADD45A, MET, MCL1, SOCS3	7
31	Ke-Ying Fang et al., 2021 [41]	IL6, FN1, CXCL1, CCL5, CCL2, CXCL10, EGF, FGF2, ICAM1, CXCL8, IL1B, MMP9	12
32	Mostafa Rezaei-Tavirani et al., 2021 [42]	FGA, FGG, FGF, ORM1, ORM2, PPBP, PF4, CRP, APOA2, SAA1, ACTB, CFB, LCAT, CETP, TLN1, SAA2, FGL1, CFI, YWHAZ, YWHAE, AZGP1, S100A8, CFHR1, CFHR3, PON3, PRDX6, ARHGDIB, TAGLN2, TRIM33, TUBB1, SH3BGRL3	31
33	Shenglong Li et al., 2020 [43]	IL1b and IL6	2
34	Suresh Kumar et al., 2020 [44]	VEGFA, TNF, IL-6, CXCL8, IL-10, CCL2, IL1B, TLR4, ICAM1, MMP9	10
35	Yi-Wei Zhu et al., 2020 [45]	RELA, TNF, IL6, IL1B, MAPK14, TP53, CXCL8, MAPK3, MAPK1, IL4, MAPK8, CASP8 and STAT1	13
36	Z. Bao et al., 2021 [46]	CCL11, TNFAIP6, AGTR2, FGA, CRM2, HBB, IRF1, IL1RN, IDO1, ATF3, CRM1, CCL4L1, CD163, FGG, CCL21, CCL3, SELE, CCL19, HSP90AA1, CX3CL1, SERPINA1, CSF3, THBS1, HP, SERPNE1, VCAM1, CXCL9, CCL4, PTGS2, CXCL10, CCL2, CXCL8, ALB, IL6	34
37	Zhen-Zhen Wang et al., 2021 [47]	TNF, EGFR, CASP9, EGFA, NFKB1, TP53, IL6, CASP3, MAPK8, PTGS2, GAPDH, CCL2, NFKBIA, MMP9, MMP2, CCND1, MCL1, MAPK1, MYC, CXCL8, JUN, CASP8, PPARG, IL1B	24
38	Auwul et al., 2021 [48]	PLK1, AURKB, AURKA, CDK1, CDC20, KIF11, CCNB1, KIF2C, DTL and CDC6	10
39	Mosharaf et al., 2022 [49]	TLR2, USP53, GUCY1A2, SNRPD2, NEDD9, IGF2, CXCL2, KLF6, PAG1 and ZFP36	10
40	Lee H et al., 2021 [50]	SLC3A2, SLC2A3, FOLR2, CCR1, FPR1, GPR183, CD68, FCGR3B, KLRD1, CD3D, KRT7, TPPP3, CD6, HBB, PPBP and MS4A1	16
41	Alanazi et al., 2022 [51]	NSP1, NSP3, NSP5, NSP9, NSP12, NSP13, NSP15, 3a, S, E, M, 6, 7a and N	14
Common genes in at least 5 articles		CXCL8, IL6, TNF, TP53, IL1B, MMP9, NFKBIA, PTGS2, ICAM1, STAT1, CCL2	11
Common genes in at least 6 articles		CXCL8, IL6, TNF, TP53, IL1B, MMP9	***6***
Common genes in at least 7 articles		CXCL8, IL6, TNF, TP53, IL1B	***5***
Common genes in at least 9 articles		CXCL8, IL6, TNF	***3***
Common genes in at least 11 articles		CXCL8, IL6	***2***

**Table 2:** The top 10 significantly enriched GO terms for each of BPs, MFs, and CCs based on the hHub-DEGs-set enrichment analysis, where hHub-DEGs-set consisted of 14 genes.

Source	GO Term ID	Description	*P_adj_-*Value	Gene Count	Enriched Genes
GO:MF	GO:0019899	enzyme binding	0.00000000	11	AKT1, APP, EGFR, INS, JUN, MAPK1, STAT3, TNF, TP53, UBA52, UBC
	GO:0098772	molecular function regulator activity	0.00000000	10	AKT1, APP, CXCL8, EGFR, IL6, INS, JUN, TNF, TP53, VEGFA
	GO:0042802	identical protein binding	0.00000000	10	AKT1, APP, EGFR, INS, JUN, MAPK1, STAT3, TNF, TP53, VEGFA
	GO:0005102	signaling receptor binding	0.00000000	9	APP, CXCL8, EGFR, IL6, INS, STAT3, TNF, TP53, VEGFA
	GO:0019902	phosphatase binding	0.00000003	5	AKT1, EGFR, MAPK1, STAT3, TP53
	GO:0030546	signaling receptor activator activity	0.00000003	6	APP, CXCL8, IL6, INS, TNF, VEGFA
	GO:0005515	protein binding	0.00000008	14	AKT1, APP, CXCL8, EGFR, IL6, INS, JUN, MAPK1, STAT3, TNF, TP53, UBA52, UBC, VEGFA
	GO:0005126	cytokine receptor binding	0.00000010	5	CXCL8, IL6, STAT3, TNF, VEGFA
	GO:0031625	ubiquitin protein ligase binding	0.00000013	5	EGFR, JUN, TP53, UBA52, UBC
	GO:0044389	ubiquitin-like protein ligase binding	0.00000015	5	EGFR, JUN, TP53, UBA52, UBC
GO:BP	GO:0031328	positive regulation of cellular biosynthetic process	0.00000000	14	AKT1, APP, CXCL8, EGFR, IL6, INS, JUN, MAPK1, STAT3, TNF, TP53, UBA52, UBC, VEGFA
	GO:0051090	regulation of DNA-binding transcription factor activity	0.00000000	11	AKT1, APP, IL6, INS, JUN, MAPK1, STAT3, TNF, UBA52, UBC, VEGFA
	GO:0009891	positive regulation of biosynthetic process	0.00000000	14	AKT1, APP, CXCL8, EGFR, IL6, INS, JUN, MAPK1, STAT3, TNF, TP53, UBA52, UBC, VEGFA
	GO:0001934	positive regulation of protein phosphorylation	0.00000000	12	AKT1, APP, EGFR, IL6, INS, MAPK1, STAT3, TNF, TP53, UBA52, UBC, VEGFA
	GO:0042327	positive regulation of phosphorylation	0.00000000	12	AKT1, APP, EGFR, IL6, INS, MAPK1, STAT3, TNF, TP53, UBA52, UBC, VEGFA
	GO:0010562	positive regulation of phosphorus metabolic process	0.00000000	12	AKT1, APP, EGFR, IL6, INS, MAPK1, STAT3, TNF, TP53, UBA52, UBC, VEGFA
	GO:0045937	positive regulation of phosphate metabolic process	0.00000000	12	AKT1, APP, EGFR, IL6, INS, MAPK1, STAT3, TNF, TP53, UBA52, UBC, VEGFA
	GO:0031401	positive regulation of protein modification process	0.00000000	12	AKT1, APP, EGFR, IL6, INS, MAPK1, STAT3, TNF, TP53, UBA52, UBC, VEGFA
	GO:0009719	response to endogenous stimulus	0.00000000	13	AKT1, APP, CXCL8, EGFR, IL6, INS, JUN, MAPK1, STAT3, TNF, TP53, UBA52, UBC
	GO:0071310	cellular response to organic substance	0.00000000	14	AKT1, APP, CXCL8, EGFR, IL6, INS, JUN, MAPK1, STAT3, TNF, TP53, UBA52, UBC, VEGFA
GO:CC	GO:0043233	organelle lumen	0.00000000	12	AKT1, APP, EGFR, IL6, INS, JUN, MAPK1, STAT3, TP53, UBA52, UBC, VEGFA
	GO:0070013	intracellular organelle lumen	0.00000000	12	AKT1, APP, EGFR, IL6, INS, JUN, MAPK1, STAT3, TP53, UBA52, UBC, VEGFA
	GO:0031974	membrane-enclosed lumen	0.00000000	12	AKT1, APP, EGFR, IL6, INS, JUN, MAPK1, STAT3, TP53, UBA52, UBC, VEGFA
	GO:0016020	membrane	0.00000004	13	AKT1, APP, EGFR, IL6, INS, JUN, MAPK1, STAT3, TNF, TP53, UBA52, UBC, VEGFA
	GO:0005768	endosome	0.00000005	7	APP, EGFR, INS, MAPK1, TNF, UBA52, UBC
	GO:0005783	endoplasmic reticulum	0.00000012	8	APP, EGFR, IL6, INS, MAPK1, TP53, UBA52, UBC
	GO:0071944	cell periphery	0.00000012	11	AKT1, APP, EGFR, IL6, JUN, MAPK1, STAT3,TNF, UBA52, UBC, VEGFA
	GO:0005576	extracellular region	0.00000013	10	APP, CXCL8, EGFR, IL6, INS, MAPK1, TNF, UBA52, UBC, VEGFA
	GO:0012505	endomembrane system	0.00000014	10	APP, EGFR, IL6, INS, MAPK1, TNF, TP53, UBA52, UBC, VEGFA
	GO:0031983	vesicle lumen	0.00000017	5	APP, EGFR, INS, MAPK1, VEGFA

**Table 3:** Interacting properties of top-ranked three drug-target complexes. The fourth, fifth, and 6th columns displayed the surface view, binding poses, interacting mode, respectively. The 7th, 8th, and 9th columns showed the interacting residues, bonding types, and distances, respectively.

Potential Targets	Structure of Ligand	Binding Affinity (kCal/mol)	Surface View of Complex	Pose View of Complex	Target Ligand Interaction	Interacting Amino Acid	Bond Type	Distance (A^0^)
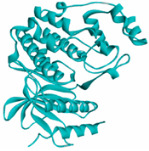 MAPK1	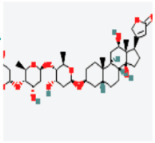 Digoxin	−11.0	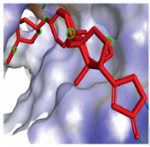	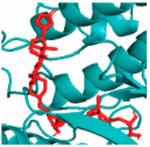	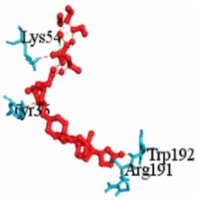	ARG191 TRP192 ALA52 LUE170 VAL39 LUE56 ILE84	CH CH A A A A A	2.55 2.783 3.544 4.820 5.029 4.883 4.404
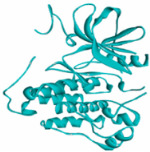 EGFR	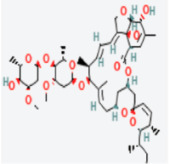 Avermectin	−10.8	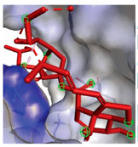	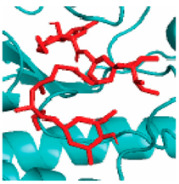	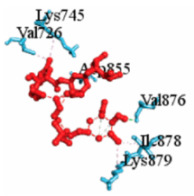	VAL876 ASP855 VAL726 LYS745 ILE878 LYS878 ARG858 PHE723	CH CHB A A A A A PA	2.068 3.581 4.181 4.063 5.221 4.334 5.086 4.743
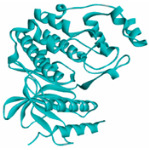 MAPK1	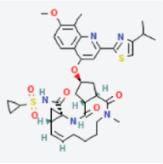 Simprevir	−10.3	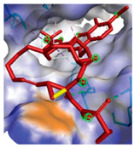	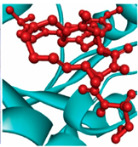	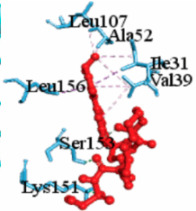	LYS151 SER153 ILE31 LEU156 ALA52 VAL39 LYS54 LEU107 LEU156	CH CH PS PS A A A A A	2.176 2.985 3.804 3.638 3.738 4.700 4.539 4.940 4.662

## Data Availability

All data analyzed in this study was provided in Table 1 and Table S1.

## Supplementary Materials

The following supporting information can be downloaded at: https://www.mdpi.com/article/10.3390/vaccines10081248/s1, Supplementary Table S1: Basic characteristics/information of the selected articles. Supplementary Table S2: (a): 2D structures of the proposed drug agents/molecules. (b) Tanimoto’s drug/chemical similarity coefficients.; Supplementary File S1: Total 145 drugs/drug chemical list that was used in this study for molecular docking.; Supplementary File S2: Molecular docking scores of top 14 proposed hHub-DEGs and the 145 drug elements.; Supplementary File S3: Molecular docking scores of top 11 published hub-DEGs and the 145 drug elements.; Supplementary Figure S1: Docking poses of the proposed 10 ligands with the proposed top ordered 6 targets (a) MAPK1, (b) EGFR, (c) CXCL8, (d) STAT3, € UBC7, and (f) TP53.; Supplementary Figure S2: Docking poses of the proposed 10 ligands with the previously published top ordered 4 targets (uncommon) (a) NFKBIA, (b) IRF7, (c) MX1, and (d) CASP3.; Supplementary Figure S3: Survival curve analysis of the proposed hHub-DEGs.

## References

[1] Zhu R.F., Gao Y.L., Robert S.H., Gao J.P., Yang S.G., Zhu C.T. (2020). Systematic Review of the Registered Clinical Trials for Coronavirus Disease 2019 (COVID-19). J. Transl. Med..

[2] Treatments and Vaccines for COVID-19|European Medicines Agency. https://www.ema.europa.eu/en/human-regulatory/overview/public-health-threats/coronavirus-disease-covid-19/treatments-vaccines-covid-19#authorised-medicines-section.

[3] Santiago J.A., Bottero V., Potashkin J.A. (2017). Dissecting the Molecular Mechanisms of Neurodegenerative Diseases through Network Biology. Front. Aging Neurosci..

[4] Rahman M.R., Islam T., Turanli B., Zaman T., Faruquee H.M., Rahman M.M., Mollah M.N.H., Nanda R.K., Arga K.Y., Gov E. (2019). Network-Based Approach to Identify Molecular Signatures and Therapeutic Agents in Alzheimer’s Disease. Comput. Biol. Chem..

[5] Islam T., Rahman R., Gov E., Turanli B., Gulfidan G., Haque A., Arga K.Y., Haque Mollah N. (2018). Drug Targeting and Biomarkers in Head and Neck Cancers: Insights from Systems Biology Analyses. Omi. A J. Integr. Biol..

[6] Shahjaman M., Rezanur Rahman M., Shahinul Islam S.M., Nurul Haque Mollah M. (2019). A Robust Approach for Identification of Cancer Biomarkers and Candidate Drugs. Medicina.

[7] Moni M.A., Islam M.B., Rahman M.R., Rashed-Al-Mahfuz M., Awal M.A., Islam S.M.S., Mollah M.N.H., Quinn J.M.W. (2020). Network-Based Computational Approach to Identify Delineating Common Cell Pathways Influencing Type 2 Diabetes and Diseases of Bone and Joints. IEEE Access.

[8] Xie T.A., Han M.Y., Su X.R., Li H.H., Chen J.C., Guo X.G. (2020). Identification of Hub Genes Associated with Infection of Three Lung Cell Lines by SARS-CoV-2 with Integrated Bioinformatics Analysis. J. Cell. Mol. Med..

[9] Oh J.H., Tannenbaum A., Deasy J.O. (2020). Identification of Biological Correlates Associated with Respiratory Failure in COVID-19. BMC Med. Genom..

[10] Ge C., He Y. (2020). In Silico Prediction of Molecular Targets of Astragaloside IV for Alleviation of COVID-19 Hyperinflammation by Systems Network Pharmacology and Bioinformatic Gene Expression Analysis. Front. Pharmacol..

[11] Aishwarya S., Gunasekaran K., Margret A.A. (2022). Computational Gene Expression Profiling in the Exploration of Biomarkers, Non-Coding Functional RNAs and Drug Perturbagens for COVID-19. J. Biomol. Struct. Dyn..

[12] Saxena A., Chaudhary U., Bharadwaj A., Wahi N., Kalli J.R., Gupta S., Kumar S., Gupta S., Raj U. (2020). A Lung Transcriptomic Analysis for Exploring Host Response in COVID-19. J. Pure Appl. Microbiol..

[13] Tao Q., Du J., Li X., Zeng J., Tan B., Xu J., Lin W., Chen X. (2020). lin Network Pharmacology and Molecular Docking Analysis on Molecular Targets and Mechanisms of Huashi Baidu Formula in the Treatment of COVID-19. Drug Dev. Ind. Pharm..

[14] Zhang N., Zhao Y.D., Wang X.M. (2020). CXCL10 an Important Chemokine Associated with Cytokine Storm in COVID-19 Infected Patients. Eur. Rev. Med. Pharmacol. Sci..

[15] Han L., Wei X.X., Zheng Y.J., Zhang L.L., Wang X.M., Yang H.Y., Ma X., Zhao L.H., Tong X.L. (2020). Potential Mechanism Prediction of Cold-Damp Plague Formula against COVID-19 via Network Pharmacology Analysis and Molecular Docking. Chin. Med..

[16] Wang Z., Jiang C., Zhang X., Zhang Y., Ren Y. (2020). Identication of Key Genes and Pathways in SARS-CoV-2 Infection Using Bioinformatics Analysis. Res. Sq..

[17] Gu H., Yuan G. (2020). Identification of Potential Key Genes for SARS-CoV-2 Infected Human Bronchial Organoids Based on Bioinformatics Analysis. bioRxiv.

[18] Soon Nan K., Karuppanan K., Kumar S., Alam S. (2021). Identification of Common Key Genes and Pathways between COVID-19 and Lung Cancer by Using Protein-Protein Interaction Network Analysis. bioRxiv.

[19] Sardar R., Satish D., Gupta D. (2020). Identification of Novel SARS-CoV-2 Drug Targets by Host MicroRNAs and Transcription Factors Co-Regulatory Interaction Network Analysis. Front. Genet..

[20] Vastrad B., Vastrad C., Tengli A. (2020). Identification of Potential MRNA Panels for Severe Acute Respiratory Syndrome Coronavirus 2 (COVID-19) Diagnosis and Treatment Using Microarray Dataset and Bioinformatics Methods. 3 Biotech.

[21] Chong X., Peng R., Sun Y., Zhang L., Zhang Z. (2020). Identification of Key Genes in Gastric Cancer by Bioinformatics Analysis. Biomed Res. Int..

[22] Prasad K., Khatoon F., Rashid S., Ali N., AlAsmari A.F., Ahmed M.Z., Alqahtani A.S., Alqahtani M.S., Kumar V. (2020). Targeting Hub Genes and Pathways of Innate Immune Response in COVID-19: A Network Biology Perspective. Int. J. Biol. Macromol..

[23] Satu S., Khan I., Rahman R., Howlader K.C., Roy S., Roy S.S., Quinn J.M.W., Moni M.A. (2021). Diseasome and Comorbidities Complexities of SARS-CoV-2 Infection with Common Malignant Diseases. Brief. Bioinform..

[24] Taz T.A., Ahmed K., Paul B.K., Kawsar M., Aktar N., Mahmud S.M.H., Moni M.A. (2021). Network-Based Identification Genetic Effect of SARS-CoV-2 Infections to Idiopathic Pulmonary Fibrosis (IPF) Patients. Brief. Bioinform..

[25] Moni M.A., Quinn J.M.W., Sinmaz N., Summers M.A. (2020). Gene Expression Profiling of SARS-CoV-2 Infections Reveal Distinct Primary Lung Cell and Systemic Immune Infection Responses That Identify Pathways Relevant in COVID-19 Disease. Brief. Bioinform..

[26] Islam T., Rahman M.R., Aydin B., Beklen H., Arga K.Y., Shahjaman M. (2020). Integrative Transcriptomics Analysis of Lung Epithelial Cells and Identification of Repurposable Drug Candidates for COVID-19. Eur. J. Pharmacol..

[27] Zhou Y., Hou Y., Shen J., Huang Y., Martin W., Cheng F. (2020). Network-Based Drug Repurposing for Novel Coronavirus 2019-NCoV/SARS-CoV-2. Cell Discov..

[28] Caradonna A., Patel T., Toleska M., Alabed S., Chang S.L. (2022). Meta-Analysis of APP Expression Modulated by SARS-CoV-2 Infection via the ACE2 Receptor. Int. J. Mol. Sci..

[29] Gu H., Yuan G. (2020). Identification of Key Genes in SARS-CoV-2 Patients on Bioinformatics Analysis. bioRxiv.

[30] Gu H., Yuan G. (2020). Identification of Key Genes and Pathways in the HPSC-Derived Lungs Infected by the SARS-CoV-2. Res. Sq..

[31] Selvaraj G., Kaliamurthi S., Peslherbe G.H., Wei D.Q. (2021). Identifying Potential Drug Targets and Candidate Drugs for COVID-19: Biological Networks and Structural Modeling Approaches. F1000Research.

[32] Tian J., Sun D., Xie Y., Liu K., Ma Y. (2020). Network Pharmacology-Based Study of the Molecular Mechanisms of Qixuekang in Treating COVID-19 during the Recovery Period. Int. J. Clin. Exp. Pathol..

[33] Jha P.K., Vijay A., Halu A., Uchida S., Aikawa M. (2021). Gene Expression Profiling Reveals the Shared and Distinct Transcriptional Signatures in Human Lung Epithelial Cells Infected With SARS-CoV-2, MERS-CoV, or SARS-CoV: Potential Implications in Cardiovascular Complications of COVID-19. Front. Cardiovasc. Med..

[34] Ramesh P., Veerappapillai S., Karuppasamy R. (2021). Gene Expression Profiling of Corona Virus Microarray Datasets to Identify Crucial Targets in COVID-19 Patients. Gene Rep..

[35] Li Z., Yang L. (2020). Underlying Mechanisms and Candidate Drugs for COVID-19 Based on the Connectivity Map Database. Front. Genet..

[36] Li G., He X., Zhang L., Ran Q., Wang J., Xiong A., Wu D., Chen F., Sun J., Chang C. (2020). Assessing ACE2 Expression Patterns in Lung Tissues in the Pathogenesis of COVID-19. J. Autoimmun..

[37] Prasad K., AlOmar S.Y., Alqahtani S.A.M., Malik M.Z., Kumar V. (2021). Brain Disease Network Analysis to Elucidate the Neurological Manifestations of COVID-19. Mol. Neurobiol..

[38] Liu F., Li Y., Yang Y., Li M., Du Y., Zhang Y., Wang J., Shi Y. (2021). Study on Mechanism of Matrine in Treatment of COVID-19 Combined with Liver Injury by Network Pharmacology and Molecular Docking Technology. Drug Deliv..

[39] Nain Z., Rana H.K., Liò P., Islam S.M.S., Summers M.A., Moni M.A. (2021). Pathogenetic Profiling of COVID-19 and SARS-like Viruses. Brief. Bioinform..

[40] Fang K.Y., Cao W.C., Xie T.A., Lv J., Chen J.X., Cao X.J., Li Z.W., Deng S.T., Guo X.G. (2021). Exploration and Validation of Related Hub Gene Expression during SARS-CoV-2 Infection of Human Bronchial Organoids. Hum. Genom..

[41] Rezaei-Tavirani M., Nejad M.R., Arjmand B., Tavirani S.R., Razzaghi M., Mansouri V. (2021). Fibrinogen Dysregulation Is a Prominent Process in Fatal Conditions of COVID-19 Infection; a Proteomic Analysis. Arch. Acad. Emerg. Med..

[42] Li S., Wang W., Li T., Han X., Hu C., Wang Y., Shen M., Du L., Nai Y., Wang J. (2022). Immune Characteristics Analysis Reveals Two Key Inflammatory Factors Correlated to the Expressions of SARS-CoV-2 S1-Specific Antibodies. Genes Dis..

[43] Kumar S. (2020). COVID-19: A Drug Repurposing and Biomarker Identification by Using Comprehensive Gene-Disease Associations through Protein-Protein Interaction Network Analysis. Preprints.

[44] Zhu Y.W., Yan X.F., Ye T.J., Hu J., Wang X.L., Qiu F.J., Liu C.H., Hu X.D. (2021). Analyzing the Potential Therapeutic Mechanism of Huashi Baidu Decoction on Severe COVID-19 through Integrating Network Pharmacological Methods. J. Tradit. Complement. Med..

[45] Bao Z., Wang L.J., He K., Lin X., Yu T., Li J., Gong J. (2021). Xiang High Expression of Ace2 in the Human Lung Leads to the Release of Il6 by Suppressing Cellular Immunity: Il6 Plays a Key Role in COVID-19. Eur. Rev. Med. Pharmacol. Sci..

[46] Wang Z.Z., Li K., Maskey A.R., Huang W., Toutov A.A., Yang N., Srivastava K., Geliebter J., Tiwari R., Miao M. (2021). A Small Molecule Compound Berberine as an Orally Active Therapeutic Candidate against COVID-19 and SARS: A Computational and Mechanistic Study. FASEB J..

[47] Auwul M.R., Rahman M.R., Gov E., Shahjaman M., Moni M.A. (2021). Bioinformatics and Machine Learning Approach Identifies Potential Drug Targets and Pathways in COVID-19. Brief. Bioinform..

[48] Mosharaf M.P., Reza M.S., Kibria M.K., Ahmed F.F., Kabir M.H., Hasan S., Mollah M.N.H. (2022). Computational Identification of Host Genomic Biomarkers Highlighting Their Functions, Pathways and Regulators That Influence SARS-CoV-2 Infections and Drug Repurposing. Sci. Rep..

[49] Lee H., Park J., Im H.J., Na K.J., Choi H. (2021). Discovery of Potential Imaging and Therapeutic Targets for Severe Inflammation in COVID-19 Patients. Sci. Rep..

[50] Alanazi K.M., Farah M.A., Hor Y.Y. (2022). Multi-Targeted Approaches and Drug Repurposing Reveal Possible SARS-CoV-2 Inhibitors. Vaccines.

[51] Beck B.R., Shin B., Choi Y., Park S., Kang K. (2020). Predicting Commercially Available Antiviral Drugs That May Act on the Novel Coronavirus (SARS-CoV-2) through a Drug-Target Interaction Deep Learning Model. Comput. Struct. Biotechnol. J..

[52] Xia J., Gill E.E., Hancock R.E.W. (2015). NetworkAnalyst for Statistical, Visual and Network-Based Meta-Analysis of Gene Expression Data. Nat. Protoc..

[53] Boyle E.I., Weng S., Gollub J., Jin H., Botstein D., Cherry J.M., Sherlock G. (2004). GO::TermFinder-Open Source Software for Accessing Gene Ontology Information and Finding Significantly Enriched Gene Ontology Terms Associated with a List of Genes INTRODUCTION: MOTIVATION AND DESIGN. Bioinforma. Appl. NOTE.

[54] Szklarczyk D., Morris J.H., Cook H., Kuhn M., Wyder S., Simonovic M., Santos A., Doncheva N.T., Roth A., Bork P. (2017). The STRING Database in 2017: Quality-Controlled Protein-Protein Association Networks, Made Broadly Accessible. Nucleic Acids Res..

[55] Kanehisa M., Furumichi M., Tanabe M., Sato Y., Morishima K. (2017). KEGG: New Perspectives on Genomes, Pathways, Diseases and Drugs. Nucleic Acids Res..

[56] Kanehisa M., Sato Y., Kawashima M., Furumichi M., Tanabe M. (2016). KEGG as a Reference Resource for Gene and Protein Annotation. Nucleic Acids Res..

[57] Kanehisa M., Goto S. (2000). KEGG: Kyoto Encyclopedia of Genes and Genomes. Nucleic Acids Res..

[58] Benjamini Y., Hochberg Y. (1995). Controlling the False Discovery Rate: A Practical and Powerful Approach to Multiple Testing. J. R. Stat. Soc. Ser. B.

[59] Khan A., Fornes O., Stigliani A., Gheorghe M., Castro-Mondragon J.A., Van Der Lee R., Bessy A., Chèneby J., Kulkarni S.R., Tan G. (2018). JASPAR 2018: Update of the Open-Access Database of Transcription Factor Binding Profiles and Its Web Framework. Nucleic Acids Res..

[60] Karagkouni D., Paraskevopoulou M.D., Chatzopoulos S., Vlachos I.S., Tastsoglou S., Kanellos I., Papadimitriou D., Kavakiotis I., Maniou S., Skoufos G. (2018). DIANA-TarBase v8: A Decade-Long Collection of Experimentally Supported MiRNA-Gene Interactions. Nucleic Acids Res..

[61] Aguirre-Gamboa R., Gomez-Rueda H., Martínez-Ledesma E., Martínez-Torteya A., Chacolla-Huaringa R., Rodriguez-Barrientos A., Tamez-Peña J.G., Treviño V. (2013). SurvExpress: An Online Biomarker Validation Tool and Database for Cancer Gene Expression Data Using Survival Analysis. PLoS ONE.

[62] Berman H.M., Westbrook J., Feng Z., Gilliland G., Bhat T.N., Weissig H., Shindyalov I.N., Bourne P.E. (2000). The Protein Data Bank. Nucleic Acids Res..

[63] Waterhouse A., Bertoni M., Bienert S., Studer G., Tauriello G., Gumienny R., Heer F.T., De Beer T.A.P., Rempfer C., Bordoli L. (2018). SWISS-MODEL: Homology Modelling of Protein Structures and Complexes. Nucleic Acids Res..

[64] Kim S., Chen J., Cheng T., Gindulyte A., He J., He S., Li Q., Shoemaker B.A., Thiessen P.A., Yu B. (2019). PubChem 2019 Update: Improved Access to Chemical Data. Nucleic Acids Res..

[65] Discovery Studio (2014). Discovery Studio Visualizer. Discovery.

[66] Morris G.M., Ruth H., Lindstrom W., Sanner M.F., Belew R.K., Goodsell D.S., Olson A.J. (2009). Software News and Updates AutoDock4 and AutoDockTools4: Automated Docking with Selective Receptor Flexibility. J. Comput. Chem..

[67] Hanwell M.D., Curtis D.E., Lonie D.C., Vandermeerschd T., Zurek E., Hutchison G.R. (2012). Avogadro: An Advanced Semantic Chemical Editor, Visualization, and Analysis Platform. J. Cheminform..

[68] Trott O., Olson A.J. (2009). AutoDock Vina: Improving the Speed and Accuracy of Docking with a New Scoring Function, Efficient Optimization, and Multithreading. J. Comput. Chem..

[69] Delano W.L., Bromberg S. (2004). PyMOL User’s Guide.

[70] The UniProt Consortium (2019). UniProt: A Worldwide Hub of Protein Knowledge. Nucleic Acids Res..

[71] Tanimoto T. (1958). An Elementary Mathematical Theory of Classification and Prediction.

[72] Willett P., Barnard J.M., Downs G.M. (1998). Chemical Similarity Searching. J. Chem. Inf. Comput. Sci..

[73] Carpten J.D., Faber A.L., Horn C., Donoho G.P., Briggs S.L., Robbins C.M., Hostetter G., Boguslawski S., Moses T.Y., Savage S. (2007). A Transforming Mutation in the Pleckstrin Homology Domain of AKT1 in Cancer. Nature.

[74] Emamian E.S. (2012). AKT/GSK3 Signaling Pathway and Schizophrenia. Front. Mol. Neurosci..

[75] Lindhurst M.J., Sapp J.C., Teer J.K., Johnston J.J., Finn E.M., Peters K., Turner J., Cannons J.L., Bick D., Blakemore L. (2011). A Mosaic Activating Mutation in AKT1 Associated with the Proteus Syndrome. N. Engl. J. Med..

[76] Schwab S.G., Hoefgen B., Hanses C., Hassenbach M.B., Albus M., Lerer B., Trixler M., Maier W., Wildenauer D.B. (2005). Further Evidence for Association of Variants in the AKT1 Gene with Schizophrenia in a Sample of European Sib-Pair Families. Biol. Psychiatry.

[77] Bai X., Tang Y., Li Q., Chen Y., Liu D., Liu G., Fan X., Ma R., Wang S., Li L. (2021). Network Pharmacology Integrated Molecular Docking Reveals the Bioactive Components and Potential Targets of Morinda Officinalis–Lycium Barbarum Coupled-Herbs against Oligoasthenozoospermia. Sci. Rep..

[78] Gassen N.C., Niemeyer D., Muth D., Corman V.M., Martinelli S., Gassen A., Hafner K., Papies J., Mösbauer K., Zellner A. (2019). SKP2 Attenuates Autophagy through Beclin1-Ubiquitination and Its Inhibition Reduces MERS-Coronavirus Infection. Nat. Commun..

[79] Khan A.A., Khan Z. (2020). System Biological Investigations of Hydroxychloroquine and Azithromycin Targets and Their Implications in QT Interval Prolongation. Chem. Biol. Interact..

[80] Fraiman P., Freire M., Moreira-Neto M., Godeiro-Junior C. (2020). Hemorrhagic Stroke and COVID-19 Infection: Coincidence or Causality?. eNeurologicalSci.

[81] XQ C., Z X., QD C., RJ S., X Z., B T., WC M., YE Y. (2021). Mechanistic Analysis of Age-Related Clinical Manifestations in Down Syndrome. Front. Aging Neurosci..

[82] Bie Y., Ge W., Yang Z., Cheng X., Zhao Z., Li S., Wang W., Wang Y., Zhao X., Yin Z. (2019). The Crucial Role of CXCL8 and Its Receptors in Colorectal Liver Metastasis. Dis. Markers.

[83] Metzemaekers M., Vandendriessche S., Berghmans N., Gouwy M., Proost P. (2020). Truncation of CXCL8 to CXCL8(9-77) Enhances Actin Polymerization and in Vivo Migration of Neutrophils. J. Leukoc. Biol..

[84] Bethune G., Bethune D., Ridgway N., Xu Z. (2010). Epidermal Growth Factor Receptor (EGFR) in Lung Cancer: An Overview and Update. J. Thorac. Dis..

[85] Purcaru O.S., Artene S.A., Barcan E., Silosi C.A., Stanciu I., Danoiu S., Tudorache S., Tataranu L.G., Dricu A. (2021). The Interference between SARS-CoV-2 and Tyrosine Kinase Receptor Signaling in Cancer. Int. J. Mol. Sci..

[86] Hu W., Zhang S., Shen Y., Yang Q. (2018). Epidermal Growth Factor Receptor Is a Co-Factor for Transmissible Gastroenteritis Virus Entry. Virology.

[87] Hu W., Zhu L., Yang X., Lin J., Yang Q. (2016). The Epidermal Growth Factor Receptor Regulates Cofilin Activity and Promotes Transmissible Gastroenteritis Virus Entry into Intestinal Epithelial Cells. Oncotarget.

[88] Balmanno K., Cook S.J. (2009). Tumour Cell Survival Signalling by the ERK1/2 Pathway. Cell Death Differ..

[89] Vagapova E.R., Lebedev T.D., Prassolov V.S. (2021). Viral Fibrotic Scoring and Drug Screen Based on MAPK Activity Uncovers EGFR as a Key Regulator of COVID-19 Fibrosis. Sci. Rep..

[90] Channappanavar R., Zhao J., Perlman S. (2014). T Cell-Mediated Immune Response to Respiratory Coronaviruses. Immunol. Res..

[91] Wen J., Wang Y.T., Valentine K.M., dos Santos Alves R.P., Xu Z., Regla-Nava J.A., Ngono A.E., Young M.P., Ferreira L.C.S., Shresta S. (2020). CD4+ T Cells Cross-Reactive with Dengue and Zika Viruses Protect against Zika Virus Infection. Cell Rep..

[92] Maes B., Bosteels C., De Leeuw E., Declercq J., Van Damme K., Delporte A., Demeyere B., Vermeersch S., Vuylsteke M., Willaert J. (2020). Treatment of Severely Ill COVID-19 Patients with Anti-Interleukin Drugs (COV-AID): A Structured Summary of a Study Protocol for a Randomised Controlled Trial. Trials.

[93] Sinaei R., Pezeshki S., Parvaresh S., Sinaei R. (2021). Why COVID-19 Is Less Frequent and Severe in Children: A Narrative Review. World J. Pediatr..

[94] Fajgenbaum D.C. (2018). Novel Insights and Therapeutic Approaches in Idiopathic Multicentric Castleman Disease. Blood.

[95] Wang X., Miller E.B., Goswami M., Zhang P., Ronning K.E., Karlen S.J., Zawadzki R.J., Pugh E.N., Burns M.E. (2017). Rapid Monocyte Infiltration Following Retinal Detachment Is Dependent on Non-Canonical IL6 Signaling through Gp130. J. Neuroinflammation.

[96] Liu B., Li M., Zhou Z., Guan X., Xiang Y. (2020). Can We Use Interleukin-6 (IL-6) Blockade for Coronavirus Disease 2019 (COVID-19)-Induced Cytokine Release Syndrome (CRS)?. J. Autoimmun..

[97] Gubernatorova E.O., Gorshkova E.A., Polinova A.I., Drutskaya M.S. (2020). IL-6: Relevance for Immunopathology of SARS-CoV-2. Cytokine Growth Factor Rev..

[98] Han H., Ma Q., Li C., Liu R., Zhao L., Wang W., Zhang P., Liu X., Gao G., Liu F. (2020). Profiling Serum Cytokines in COVID-19 Patients Reveals IL-6 and IL-10 Are Disease Severity Predictors. Emerg. Microbes Infect..

[99] Saghazadeh A., Rezaei N. (2020). Towards Treatment Planning of COVID-19: Rationale and Hypothesis for the Use of Multiple Immunosuppressive Agents: Anti-Antibodies, Immunoglobulins, and Corticosteroids. Int. Immunopharmacol..

[100] Kaur S., Bansal Y., Kumar R., Bansal G. (2020). A Panoramic Review of IL-6: Structure, Pathophysiological Roles and Inhibitors. Bioorganic Med. Chem..

[101] Chen X., Zhao B., Qu Y., Chen Y., Xiong J., Feng Y., Men D., Huang Q., Liu Y., Yang B. (2020). Detectable Serum SARS-CoV-2 Viral Load (RNAaemia) Is Closely Correlated with Drastically Elevated Interleukin 6 (IL-6) Level in Critically Ill COVID-19 Patients. Clin. Infect. Dis..

[102] Fung T.S., Liu D.X. (2017). Activation of the C-Jun NH2-Terminal Kinase Pathway by Coronavirus Infectious Bronchitis Virus Promotes Apoptosis Independently of c-Jun Article. Cell Death Dis..

[103] Tiwari R., Mishra A.R., Gupta A., Nayak D. (2022). Structural Similarity-Based Prediction of Host Factors Associated with SARS-CoV-2 Infection and Pathogenesis. J. Biomol. Struct. Dyn..

[104] Tang Y., Geng Y., Luo J., Shen W., Zhu W., Meng C., Li M., Zhou X., Zhang S., Cao J. (2015). Downregulation of Ubiquitin Inhibits the Proliferation and Radioresistance of Non-Small Cell Lung Cancer Cells In Vitro and In Vivo. Sci. Rep..

[105] Han B., Qu Y., Jin Y., Yu Y., Deng N., Wawrowsky K., Zhang X., Li N., Bose S., Wang Q. (2015). FOXC1 Activates Smoothened-Independent Hedgehog Signaling in Basal-like Breast Cancer. Cell Rep..

[106] Haffner M.C., Weier C., Xu M.M., Vaghasia A., Gürel B., Gümüşkaya B., Esopi D.M., Fedor H., Tan H.L., Kulac I. (2016). Molecular Evidence That Invasive Adenocarcinoma Can Mimic Prostatic Intraepithelial Neoplasia (PIN) and Intraductal Carcinoma through Retrograde Glandular Colonization. J. Pathol..

[107] Yu S., Jiang X., Li J., Li C., Guo M., Ye F., Zhang M., Jiao Y., Guo B. (2019). Comprehensive Analysis of the GATA Transcription Factor Gene Family in Breast Carcinoma Using Gene Microarrays, Online Databases and Integrated Bioinformatics. Sci. Rep..

[108] Tessema M., Yingling C.M., Snider A.M., Do K., Juri D.E., Picchi M.A., Zhang X., Liu Y., Leng S., Tellez C.S. (2014). GATA2 Is Epigenetically Repressed in Human and Mouse Lung Tumors and Is Not Requisite for Survival of KRAS Mutant Lung Cancer. J. Thorac. Oncol..

[109] Huang T., Wang G., Yang L., Peng B., Wen Y., Ding G., Wang Z. (2017). Transcription Factor YY1 Modulates Lung Cancer Progression by Activating LncRNA-PVT1. DNA Cell Biol..

[110] Zhang S., Li M., Ji H., Fang Z. (2018). Landscape of Transcriptional Deregulation in Lung Cancer. BMC Genom..

[111] Lee H.K., Lee D.S., Park J.C. (2015). Nuclear Factor I-C Regulates E-Cadherin via Control of KLF4 in Breast Cancer. BMC Cancer.

[112] Marimuthu A., Jacob H.K.C., Jakharia A., Subbannayya Y., Keerthikumar S., Kashyap M.K., Goel R., Balakrishnan L., Dwivedi S., Pathare S. (2011). Gene Expression Profiling of Gastric Cancer. J. Proteom. Bioinforma..

[113] Brun M., Coles J.E., Monckton E.A., Glubrecht D.D., Bisgrove D., Godbout R. (2009). Nuclear Factor I Regulates Brain Fatty Acid-Binding Protein and Glial Fibrillary Acidic Protein Gene Expression in Malignant Glioma Cell Lines. J. Mol. Biol..

[114] Rahman M.R., Islam T., Gov E., Turanli B., Gulfidan G., Shahjaman M., Banu N.A., Mollah M.N.H., Arga K.Y., Moni M.A. (2019). Identification of Prognostic Biomarker Signatures and Candidate Drugs in Colorectal Cancer: Insights from Systems Biology Analysis. Medicina.

[115] Ma J., Wang W., Azhati B., Wang Y., Tusong H. (2020). MiR-106a-5p Functions as a Tumor Suppressor by Targeting VEGFA in Renal Cell Carcinoma. Dis. Markers.

[116] Majd M., Hosseini A., Ghaedi K., Kiani-Esfahani A., Tanhaei S., Shiralian-Esfahani H., Rahnamaee S.Y., Mowla S.J., Nasr-Esfahani M.H. (2018). MiR-9-5p and MiR-106a-5p Dysregulated in CD4+ T-Cells of Multiple Sclerosis Patients and Targeted Essential Factors of T Helper17/Regulatory T-Cells Differentiation. Iran. J. Basic Med. Sci..

[117] Liao W., He J., Disoma C., Hu Y., Li J., Chen G., Sheng Y., Cai X., Li C., Cheng K. (2021). Hsa_circ_0107593 Suppresses the Progression of Cervical Cancer via Sponging Hsa-MiR-20a-5p/93-5p/106b-5p. Front. Oncol..

[118] Fang T., Wu Q., Zhou L., Mu S., Fu Q. (2016). MiR-106b-5p and MiR-17-5p Suppress Osteogenic Differentiation by Targeting Smad5 and Inhibit Bone Formation. Exp. Cell Res..

[119] Lee J., Kim H.E., Song Y.S., Cho E.Y., Lee A. (2019). MiR-106b-5p and MiR-17-5p Could Predict Recurrence and Progression in Breast Ductal Carcinoma in Situ Based on the Transforming Growth Factor-Beta Pathway. Breast Cancer Res. Treat..

[120] Yang C., Dou R., Yin T., Ding J. (2020). MiRNA-106b-5p in Human Cancers: Diverse Functions and Promising Biomarker. Biomed. Pharmacother..

[121] Kelleci Cakir B., Bayraktar-Ekincioglu A., Demirkan K. (2021). Benefit versus Toxicity Risk of Digoxin in Patients with COVID-19. Eur. J. Hosp. Pharm..

[122] Peltzer B., Lerman B.B., Goyal P., Cheung J.W. (2021). Role for Digoxin in Patients Hospitalized with COVID-19 and Atrial Arrhythmias. J. Cardiovasc. Electrophysiol..

[123] Siniorakis E., Arvanitakis S., Katsianis A., Elkouris M. (2021). Atrial Fibrillation and Flutter in Patients Hospitalized for COVID-19: The Challenging Role of Digoxin. J. Cardiovasc. Electrophysiol..

[124] Mezaal M.H., Farhan H.A., Dakhil Z.A. (2020). COVID-19 Pandemic Impact on Physicians’ Decision-Making: Digoxin Toxicity in View of Combination of Hydroxychloroquine and Azithromycin: A Case Report. Open Access Maced. J. Med. Sci..

[125] Pandey S., Pathak S.K., Pandey A., Salunke A.A., Chawla J., Ortho M.S., Sharma A., Sharma S., Thivari P., Ratna H.V.K. (2020). Ivermectin in COVID-19: What Do We Know?. Diabetes Metab. Syndr. Clin. Res. Rev..

[126] Heidary F., Gharebaghi R. (2020). Ivermectin: A Systematic Review from Antiviral Effects to COVID-19 Complementary Regimen. J. Antibiot..

[127] Gupta D., Sahoo A.K., Singh A. (2020). Ivermectin: Potential Candidate for the Treatment of COVID 19. Braz. J. Infect. Dis..

[128] Jean S.S., Hsueh P.R. (2020). Old and Re-Purposed Drugs for the Treatment of COVID-19. Expert Rev. Anti. Infect. Ther..

[129] Hossen M.S., Barek M.A., Jahan N., Safiqul Islam M. (2020). A Review on Current Repurposing Drugs for the Treatment of COVID-19: Reality and Challenges. SN Compr. Clin. Med..

[130] Rosenquist Å., Samuelsson B., Johansson P.O., Cummings M.D., Lenz O., Raboisson P., Simmen K., Vendeville S., De Kock H., Nilsson M. (2014). Discovery and Development of Simeprevir (TMC435), a HCV NS3/4A Protease Inhibitor. J. Med. Chem..

[131] Muturi E., Hong W., Li J., Yang W., He J., Wei H., Yang H. (2022). Effects of Simeprevir on the Replication of SARS-CoV-2 in Vitro and in Transgenic HACE2 Mice. Int. J. Antimicrob. Agents.

[132] Hosseini F.S., Amanlou M. (2020). Anti-HCV and Anti-Malaria Agent, Potential Candidates to Repurpose for Coronavirus Infection: Virtual Screening, Molecular Docking, and Molecular Dynamics Simulation Study. Life Sci..

[133] Gurung A.B., Ali M.A., Lee J., Farah M.A., Al-Anazi K.M. (2021). The Potential of Paritaprevir and Emetine as Inhibitors of SARS-CoV-2 RdRp. Saudi J. Biol. Sci..

[134] Gurung A.B. (2020). In Silico Structure Modelling of SARS-CoV-2 Nsp13 Helicase and Nsp14 and Repurposing of FDA Approved Antiviral Drugs as Dual Inhibitors. Gene Rep..

