# Supplementary material for: Meta-Data Analysis to Explore the Hub of the Hub-Genes That Influence SARS-CoV-2 Infections Highlighting Their Pathogenetic Processes and Drugs Repurposing
Source: Vaccines (Basel). 2022 Aug 3;10(8):1248. doi: 10.3390/vaccines10081248 (PMC9415433; doi:10.3390/vaccines10081248)
Supplement: Supplementary file 1 [file vaccines-10-01248-s001.zip › Supplementary figures.pdf]

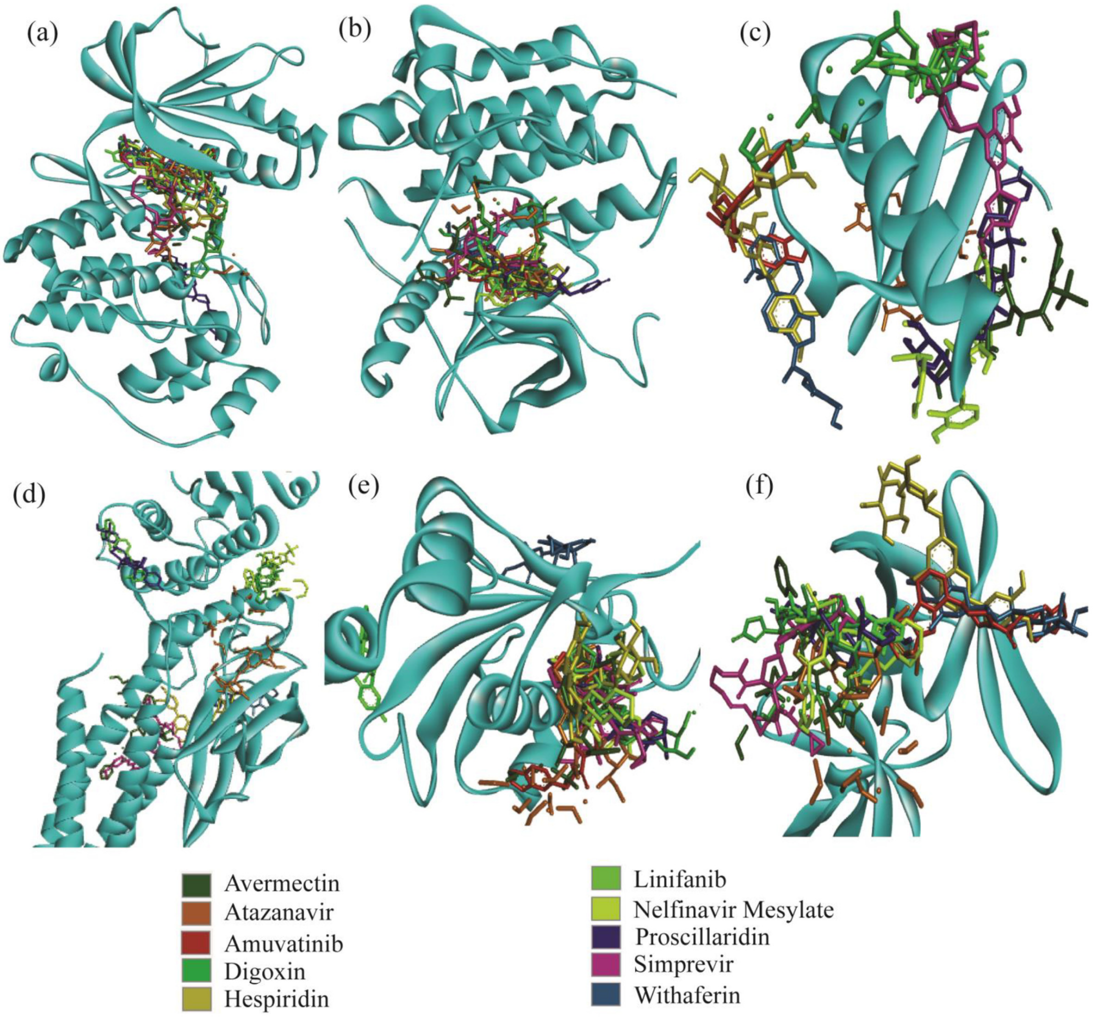

Supplementary Figure S1: Docking poses of the proposed 10 ligands with the proposed top ordered 6 targets (a) MAPK1, (b) EGFR, (c) CXCL8, (d) STAT3, (e) UBC7 and (f) TP53.

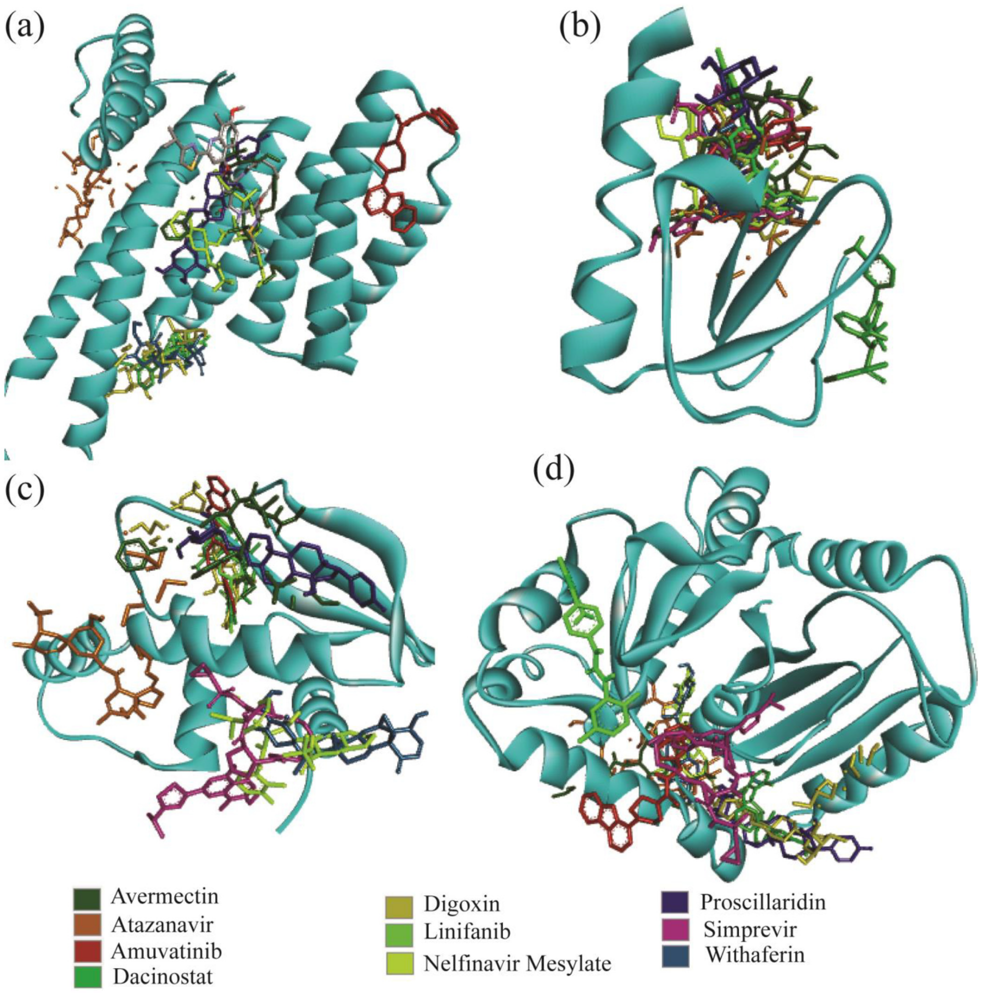

Supplementary Figure S2: Docking poses of the proposed 10 ligands with the previously published top ordered 4 targets (uncommon) (a) NFKBIA, (b) IRF7, (c) MX1 and (d) CASP3.

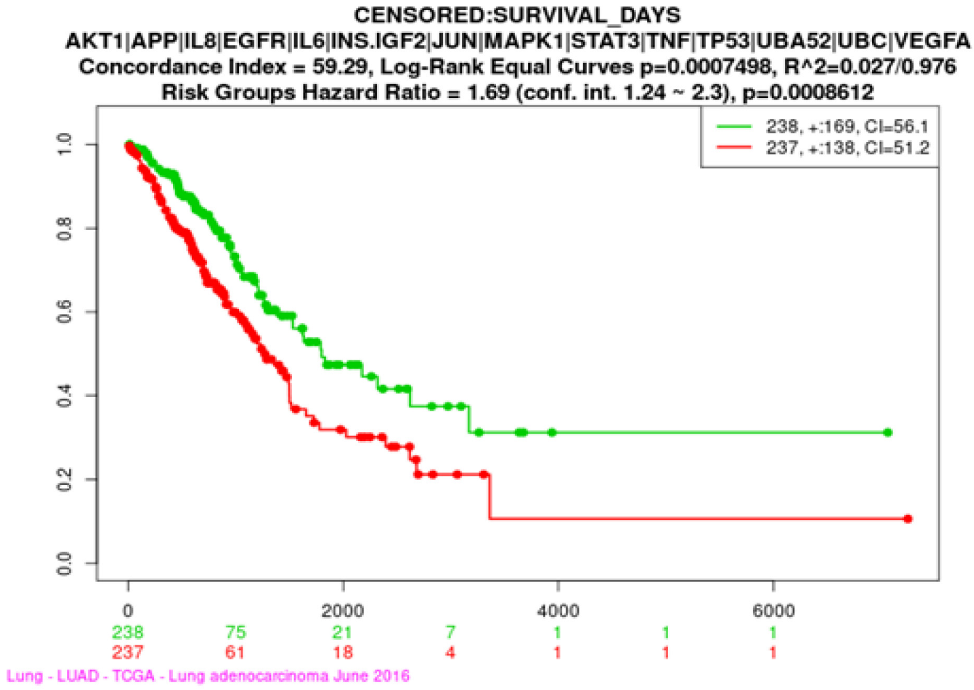

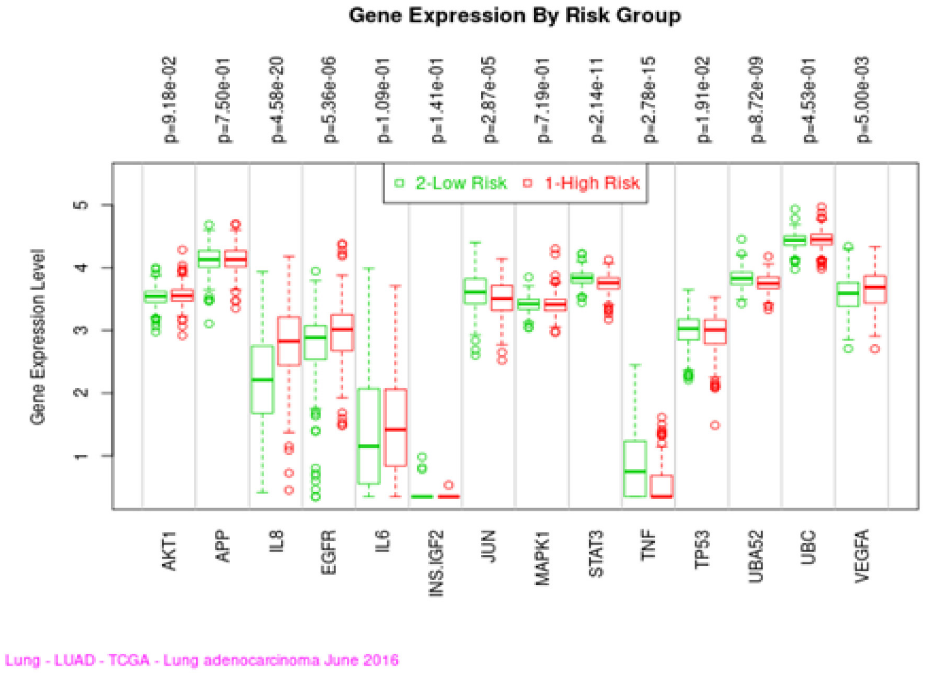

Supplementary Figure S3 : Survival curve analysis of the proposed hHub-DEGs
