# Supplementary material for: Meta-Data Analysis to Explore the Hub of the Hub-Genes That Influence SARS-CoV-2 Infections Highlighting Their Pathogenetic Processes and Drugs Repurposing
Source: Vaccines (Basel). 2022 Aug 3;10(8):1248. doi: 10.3390/vaccines10081248 (PMC9415433; doi:10.3390/vaccines10081248)
Supplement: Supplementary file 1 [file vaccines-10-01248-s001.zip › Supplementary table S1.pdf]

Supplementary Table S1: Basic characteristics/information of the selected articles.

SL	Authors	Data Types	Any comorbidity data analyzed with COVID-19 data	Statistical test for DEGs identification	Suggested drug molecules
1	Caradonna, A et al. 2022 [1]	Collected biomarker	N/A	N/A	N/A
2	Hanming Gu et al. 2020 [2]	RNA-Seq	N/A	Classical t-test	N/A
3	Kang Soon Nan et al. 2021 [3]	Collected target proteins	Lung cancer	N/A	N/A
4	Hanming Gu et al. 2020 [4]	RNA-Seq	N/A	Classical t-test	N/A
5	Rahila Sardar et al. 2020 [5]	Microarray and RNA-seq	N/A	Robust Multi-chip Average (RMA) and DESeq2	N/A
6	Hanming Gu et al. 2020 [6]	RNA-seq	N/A	Classical t-test	N/A
7	Tian-Ao Xie et al. 2020 [7]	RNA-seq	N/A	LIMMA	N/A
8	Jung Hun Oh et al. 2020 [8]	SNPs	N/A	GWAS	N/A
9	Basavaraj Vastrad et al. 2020 [9]	Microarray	N/A	LIMMA	N/A
10	Kartikay Prasad et al. 2020 [10]	RNA-Seq	N/A	DESeq2	Mitomycin-C, Imiquimod Polyinosinic:polycytidylic acid (poly I:C),S-carbamidomethylcysteine (Cysteine-S-acetamide), Vanadium oxide and MgATP
11	Gurudeeban Selvaraj et al. 2021 [11]	Microarray	N/A	LIMMA	Wortmannin
12	Md. Shahriare Satu et al. 2021 [12]	RNA-Seq and Microarray	Breast cancer, colon cancer, Kidney cancer, Liver cancer, Bladder & Prostate cancer (BPC), Thyroid cancer	linear and Bayesian method [4]	N/A
13	Tasnimul Alam Taz et al. 2020 [13]	RNA-Seq and Microarray	IPF	DESeq2 and LIMMA -GEO2R	MIGLITOL CTD 00002031; CHEMBL55802 CTD 00003118;Hesperidin CTD 00006087; Cytochalasin D CTD 00007076; Prolinedithiocarbamate CTD

					00002658; Parthenolide CTD 00000087; FEXOFENADINE HYDROCHLORIDE CTD 00003191; Hydroxytyrosol CTD 00000267; Antimycin A CTD 00005427; Anacardic acid C15:3 CTD 00003117
14	Mohammad Ali Moni et al. 2020 [14]	RNA-Seq and Microarray	SARS-CoV, MERS-CoV, and influenza A strains H1N2, H3N2, and H5N1.	DESeq2 and LIMMA	cytochalasin D; 1′-acetoxychavicol acetate; Atorvastatin; proline dithiocarbamate; dicumarol; Oleanolic acid
15	Tania Islam et al. 2020 [15]	RNA-Seq	N/A	EdgeR	SYK-inhibitor; Radicicol; Dabrafenib; AT-7519; Dasatinib; Lovastatin; Thiostrepton; Linifanib; JNK–IN–5A; Withaferin-A
16	Yadi Zhou et al. 2020 [16]	Collected protein data	SARS-CoV, MERS-CoV, HCoV-229E, and HCoV-NL63	N/A	N/A
17	Ge C et al. 2020 [17]	RNA-seq	N/A	LIMMA packages	Astragaloside IV
18	Aishwarya et al. 2020 [18]	RNA-Seq	N/A	GEO2R module of LIMMA	F-1566-0341; Digoxin; Proscillaridin; Linifanib
19	Saxena, A. et al. 2020 [19]	RNA-Seq	N/A	EdgeR	N/A
20	Tao Q et. al. 2020 [20]	Collected target proteins	N/A	N/A	Quercetin; Kaempferol; Beta-sitosterol; Stigmasterol; Isorhamnetin; Baicalein; Naringenin; Formononetin
21	Zhang N et. al. 2020 [21]	RNA Seq and Microarray	SARS -CoV	LIMMA package	N/A
22	Han L et. al. 2020 [22]	Collected target proteins	N/A	N/A	Quercetin; luteolin,
23	Tian J et al. 2020 [23]	Collected target proteins	N/A		N/A
24	Jha PK et. al. 2021 [24]	RNA-Seq and Microarray	MARS-CoV and SARS-CoV	DESeq2 and LIMMA	N/A
25	Ramesh P et al. 2020 [25]	Microarray	N/A	Student’s t-test	N/A

				and LIMMA package	
26	Li Zhonglin et al. 2020 [26]	RNA-Seq	N/A	DESeq2	Top suggested drug based on DEGs of COVID-19 patients BALF: Adiphenine, Podophyllotoxin, Amantadine, Thioperamide, Monensin, Vancomycin, Etiocholanolone, Acyclovir<br>Top suggested drug based on the genes co-expressed with ACE2: Isoflupredone, Heptaminol, Chenodeoxycholic acid, Podophyllotoxin, Atractyloside, Adiphenine, Monensin, Lisuride
27	Li G et al. 2020 [27]	Microarray	Chronic respiratory diseases including chronic obstructive pulmonary diseases (COPD) and asthma, smoker.	Gene Set Enrichment Analysis & Gene Set Variation Analysis	N/A
28	Prasad K et al. 2021 [28]	Collected target proteins	Brain related disorders	N/A	N/A
29	Fangzhou Liu et al. 2021 [29]	Collected target proteins	Liver injury	N/A	Matrine
30	Zulkar Nain et al. 2020 [30]	Microarray	SARS, Pneumonia, Diarrhea, Shortness of Breath	LIMMA	antibiotic K-252A, cabozantinib, amuvatinib, crizotinib, SGX-523, 888719-03-7, CHEMBL527066, CHEMBL503090, SCHEMBL15322421, CHEMBL462712, rac-crizotinib, CHEMBL561660, Crizotinib, Cabozantinib.
31	Ke-Ying Fang et al. 2021 [31]	RNA-Seq	N/A	Analyzed the data using the R	N/A

				package (No specific methods were mentioned)	
32	Mostafa Rezaei-Tavirani et al. 2021 [32]	Collected proteins data	N/A	N/A	N/A
33	Shenglong Li et al. 2020 [33]	RNA-Seq	Influenza HCV	DESeq2	N/A
34	Suresh Kumar et al. 2020 [34]	Collected gene name	Cough,Fever,Dyspnea Shortness,Pneumonia, Heart Disease,Kidney Disease,Lung Disease,Diabetes,Hypertension, Cancer, Lymphopenia,Pulmonary infiltrate, Leukocytosis,Neutrophilia Sepsis,Kidney injury,Coagulopathy,Thrombocytopenia Multiple organ failure,Cough,Sore throat,Runny,Diarrhea,Headache	N/A	Chloroquine, lenalidomide, Penicillin, Penicillin, Pentoxifylline, Thalidome, Sorafenib, Paclitaxel, Rapamycin, Cortisol, Statins
35	Yi-Wei Zhu et al. 2020 [35]	Collected gene name	N/A	N/A	Quercetin, Kaempferol, bsitosterol, Isorhamnetin, Naringenin, Luteolin, (þ)-catechin, Delphinidin, aloe-Emodin, Baicalein and Irisolidone
36	Z. Bao et al. 2021 [36]	RNA-Seq	N/A	LIMMA	N/A
37	Zhen-Zhen Wang et al. 2021 [37]	Collected gene name	SARS	N/A	berberine/NIT-X
38	Auwul et al. 2021 [38]	Transcriptomic data	COVID-19	LIMMA	amsacrine, BRD-K68548958, naproxol, palbociclib and teniposide
39	Mosharaf et al., 2022 [39]	Transcriptomic	COVID-19	DESeq2 and edgeR	Torin-2, Rapamycin, Radotinib,

		data			Ivermectin, Thiostrepton, Tacrolimus and Daclatasvir
40	Lee H et al., 2021 [40]	Collected marker genes	COVID-19	N/A	N/A
41	Alanazi et al., 2022 [41]	SARS-CoV-2 proteins	SARS-CoV-2	N/A	N/A

References

1. Caradonna, A.; Patel, T.; Toleska, M.; Alabed, S.; Chang, S.L. Meta-Analysis of APP Expression Modulated by SARS-CoV-2 Infection via the ACE2 Receptor. *Int. J. Mol. Sci.* **2022**, *23*, doi:10.3390/ijms23031182.
2. Gu, H.; Yuan, G. Identification of Key Genes in SARS-CoV-2 Patients on Bioinformatics Analysis. *bioRxiv* **2020**, 2020.08.09.243444, doi:10.1101/2020.08.09.243444.
3. Soon Nan, K.; Karuppanan, K.; Kumar, S.; Alam, S. Identification of Common Key Genes and Pathways between Covid-19 and Lung Cancer by Using Protein-Protein Interaction Network Analysis. *bioRxiv* **2021**, 2021.02.16.431364, doi:10.1101/2021.02.16.431364.
4. Gu, H.; Yuan, G. Identification of Key Genes and Pathways in the HPSC-Derived Lungs Infected by the SARS-CoV-2. *Prepr. (Version 1) available Res. Sq.* **2020**, 1–12.
5. Sardar, R.; Satish, D.; Gupta, D. Identification of Novel SARS-CoV-2 Drug Targets by Host MicroRNAs and Transcription Factors Co-Regulatory Interaction Network Analysis. *Front. Genet.* **2020**, *11*, doi:10.3389/fgene.2020.571274.
6. Chong, X.; Peng, R.; Sun, Y.; Zhang, L.; Zhang, Z. Identification of Key Genes in Gastric Cancer by Bioinformatics Analysis. *Biomed Res. Int.* **2020**, *2020*, doi:10.1155/2020/7658230.
7. Xie, T.A.; Han, M.Y.; Su, X.R.; Li, H.H.; Chen, J.C.; Guo, X.G. Identification of Hub Genes Associated with Infection of Three Lung Cell Lines by SARS-CoV-2 with Integrated Bioinformatics Analysis. *J. Cell. Mol. Med.* **2020**, *24*, doi:10.1111/jcmm.15862.
8. Oh, J.H.; Tannenbaum, A.; Deasy, J.O. Identification of Biological Correlates Associated with Respiratory Failure in COVID-19. *BMC Med. Genomics* **2020**, *13*, doi:10.1186/s12920-020-00839-1.
9. Vastrad, B.; Vastrad, C.; Tengli, A. Identification of Potential MRNA Panels for Severe Acute Respiratory Syndrome Coronavirus 2 (COVID-19) Diagnosis and Treatment Using Microarray Dataset and Bioinformatics Methods. *3 Biotech* **2020**, *10*, doi:10.1007/s13205-020-02406-y.
10. Prasad, K.; Khatoon, F.; Rashid, S.; Ali, N.; AlAsmari, A.F.; Ahmed, M.Z.; Alqahtani, A.S.; Alqahtani, M.S.; Kumar, V. Targeting Hub Genes and Pathways of Innate Immune Response in COVID-19: A Network Biology Perspective. *Int. J. Biol. Macromol.* **2020**, *163*, doi:10.1016/j.ijbiomac.2020.06.228.
11. Selvaraj, G.; Kaliamurthi, S.; Peslherbe, G.H.; Wei, D.Q. Identifying Potential Drug Targets and Candidate Drugs for COVID-19: Biological

Networks and Structural Modeling Approaches. *F1000Research* **2021**, *10*, 127, doi:10.12688/f1000research.50850.1.
12. Satu, S.; Khan, I.; Rahman, R.; Howlader, K.C.; Roy, S.; Roy, S.S.; Quinn, J.M.W.; Moni, M.A. Diseasome and Comorbidities Complexities of SARS-CoV-2 Infection with Common Malignant Diseases. *Brief. Bioinform.* **2021**, *22*, doi:10.1093/bib/bbab003.
13. Taz, T.A.; Ahmed, K.; Paul, B.K.; Kawsar, M.; Aktar, N.; Mahmud, S.M.H.; Moni, M.A. Network-Based Identification Genetic Effect of SARS-CoV-2 Infections to Idiopathic Pulmonary Fibrosis (IPF) Patients. *Brief. Bioinform.* **2021**, *22*, doi:10.1093/bib/bbaa235.
14. Moni, M.A.; Quinn, J.M.W.; Sinmaz, N.; Summers, M.A. Gene Expression Profiling of SARS-CoV-2 Infections Reveal Distinct Primary Lung Cell and Systemic Immune Infection Responses That Identify Pathways Relevant in COVID-19 Disease. *Brief. Bioinform.* **2020**, *2020*, 1–14, doi:10.1093/bib/bbaa376.
15. Islam, T.; Rahman, M.R.; Aydin, B.; Beklen, H.; Arga, K.Y.; Shahjaman, M. Integrative Transcriptomics Analysis of Lung Epithelial Cells and Identification of Repurposable Drug Candidates for COVID-19. *Eur. J. Pharmacol.* **2020**, *887*, doi:10.1016/j.ejphar.2020.173594.
16. Zhou, Y.; Hou, Y.; Shen, J.; Huang, Y.; Martin, W.; Cheng, F. Network-Based Drug Repurposing for Novel Coronavirus 2019-NCoV/SARS-CoV-2. *Cell Discov.* **2020**, *6*, doi:10.1038/s41421-020-0153-3.
17. Ge, C.; He, Y. In Silico Prediction of Molecular Targets of Astragaloside IV for Alleviation of COVID-19 Hyperinflammation by Systems Network Pharmacology and Bioinformatic Gene Expression Analysis. *Front. Pharmacol.* **2020**, *11*, doi:10.3389/fphar.2020.556984.
18. Aishwarya, S.; Gunasekaran, K.; Margret, A.A. Computational Gene Expression Profiling in the Exploration of Biomarkers, Non-Coding Functional RNAs and Drug Perturbagens for COVID-19. *J. Biomol. Struct. Dyn.* **2020**, doi:10.1080/07391102.2020.1850360.
19. Saxena, A.; Chaudhary, U.; Bharadwaj, A.; Wahi, N.; Kalli, J.R.; Gupta, S.; Kumar, S.; Gupta, S.; Raj, U. A Lung Transcriptomic Analysis for Exploring Host Response in COVID-19. *J. Pure Appl. Microbiol.* **2020**, *14*, doi:10.22207/JPAM.14.SPL1.47.
20. Tao, Q.; Du, J.; Li, X.; Zeng, J.; Tan, B.; Xu, J.; Lin, W.; Chen, X. lin Network Pharmacology and Molecular Docking Analysis on Molecular Targets and Mechanisms of Huashi Baidu Formula in the Treatment of COVID-19. *Drug Dev. Ind. Pharm.* **2020**, doi:10.1080/03639045.2020.1788070.
21. Zhang, N.; Zhao, Y.D.; Wang, X.M. CXCL10 an Important Chemokine Associated with Cytokine Storm in COVID-19 Infected Patients. *Eur. Rev. Med. Pharmacol. Sci.* **2020**, *24*, doi:10.26355/eurrev_202007_21922.
22. Han, L.; Wei, X.X.; Zheng, Y.J.; Zhang, L.L.; Wang, X.M.; Yang, H.Y.; Ma, X.; Zhao, L.H.; Tong, X.L. Potential Mechanism Prediction of Cold-Damp Plague Formula against COVID-19 via Network Pharmacology Analysis and Molecular Docking. *Chinese Med. (United Kingdom)* **2020**, *15*, doi:10.1186/s13020-020-00360-8.
23. Tian, J.; Sun, D.; Xie, Y.; Liu, K.; Ma, Y. Network Pharmacology-Based Study of the Molecular Mechanisms of Qixuekang in Treating COVID-19 during the Recovery Period. *Int. J. Clin. Exp. Pathol.* **2020**, *13*, 2677–2690.
24. Jha, P.K.; Vijay, A.; Halu, A.; Uchida, S.; Aikawa, M. Gene Expression Profiling Reveals the Shared and Distinct Transcriptional Signatures in Human Lung Epithelial Cells Infected With SARS-CoV-2, MERS-CoV, or SARS-CoV: Potential Implications in Cardiovascular Complications of COVID-19. *Front. Cardiovasc. Med.* **2021**, *7*, 1–15, doi:10.3389/fcvm.2020.623012.
25. Ramesh, P.; Veerappapillai, S.; Karuppasamy, R. Gene Expression Profiling of Corona Virus Microarray Datasets to Identify Crucial Targets in COVID-19 Patients. *Gene Reports* **2021**, *22*, doi:10.1016/j.genrep.2020.100980.
26. Li, Z.; Yang, L. Underlying Mechanisms and Candidate Drugs for COVID-19 Based on the Connectivity Map Database. *Front. Genet.* **2020**,

11, doi:10.3389/fgene.2020.558557.
27. Li, G.; He, X.; Zhang, L.; Ran, Q.; Wang, J.; Xiong, A.; Wu, D.; Chen, F.; Sun, J.; Chang, C. Assessing ACE2 Expression Patterns in Lung Tissues in the Pathogenesis of COVID-19. *J. Autoimmun.* **2020**, *112*, 102463, doi:10.1016/J.JAUT.2020.102463.
28. Prasad, K.; AlOmar, S.Y.; Alqahtani, S.A.M.; Malik, M.Z.; Kumar, V. Brain Disease Network Analysis to Elucidate the Neurological Manifestations of COVID-19. *Mol. Neurobiol.* **2021**, *58*, doi:10.1007/s12035-020-02266-w.
29. Liu, F.; Li, Y.; Yang, Y.; Li, M.; Du, Y.; Zhang, Y.; Wang, J.; Shi, Y. Study on Mechanism of Matrine in Treatment of COVID-19 Combined with Liver Injury by Network Pharmacology and Molecular Docking Technology. *Drug Deliv.* **2021**, *28*, 325, doi:10.1080/10717544.2021.1879313.
30. Nain, Z.; Rana, H.K.; Liò, P.; Islam, S.M.S.; Summers, M.A.; Moni, M.A. Pathogenetic Profiling of COVID-19 and SARS-like Viruses. *Brief. Bioinform.* **2021**, *22*, 1175–1196, doi:10.1093/BIB/BBAA173.
31. Fang, K.Y.; Cao, W.C.; Xie, T.A.; Lv, J.; Chen, J.X.; Cao, X.J.; Li, Z.W.; Deng, S.T.; Guo, X.G. Exploration and Validation of Related Hub Gene Expression during SARS-CoV-2 Infection of Human Bronchial Organoids. *Hum. Genomics* **2021**, *15*, 1–13, doi:10.1186/s40246-021-00316-5.
32. Rezaei-Tavirani, M.; Nejad, M.R.; Arjmand, B.; Tavirani, S.R.; Razzaghi, M.; Mansouri, V. Fibrinogen Dysregulation Is a Prominent Process in Fatal Conditions of COVID-19 Infection; a Proteomic Analysis. *Arch. Acad. Emerg. Med.* **2021**, *9*, 1–5, doi:10.22037/aaem.v9i1.1128.
33. Li, S.; Wang, W.; Li, T.; Han, X.; Hu, C.; Wang, Y.; Shen, M.; Du, L.; Nai, Y.; Wang, J.; et al. Immune Characteristics Analysis Reveals Two Key Inflammatory Factors Correlated to the Expressions of SARS-CoV-2 S1-Specific Antibodies. *Genes Dis.* **2022**, *9*, 522, doi:10.1016/J.GENDIS.2020.12.007.
34. Kumar, S. COVID-19: A Drug Repurposing and Biomarker Identification by Using Comprehensive Gene-Disease Associations through Protein-Protein Interaction Network Analysis. **2020**, doi:10.20944/PREPRINTS202003.0440.V1.
35. Zhu, Y.W.; Yan, X.F.; Ye, T.J.; Hu, J.; Wang, X.L.; Qiu, F.J.; Liu, C.H.; Hu, X.D. Analyzing the Potential Therapeutic Mechanism of Huashi Baidu Decoction on Severe COVID-19 through Integrating Network Pharmacological Methods. *J. Tradit. Complement. Med.* **2021**, *11*, doi:10.1016/j.jtcme.2021.01.004.
36. Bao, Z.; Wang, L.J.; He, K.; Lin, X.; Yu, T.; Li, J.; Gong, J.; Xiang High Expression of Ace2 in the Human Lung Leads to the Release of Il6 by Suppressing Cellular Immunity: Il6 Plays a Key Role in Covid-19. *Eur. Rev. Med. Pharmacol. Sci.* **2021**, *25*, 527–540, doi:10.26355/eurrev_202101_24425.
37. Wang, Z.Z.; Li, K.; Maskey, A.R.; Huang, W.; Toutov, A.A.; Yang, N.; Srivastava, K.; Geliebter, J.; Tiwari, R.; Miao, M.; et al. A Small Molecule Compound Berberine as an Orally Active Therapeutic Candidate against COVID-19 and SARS: A Computational and Mechanistic Study. *FASEB J.* **2021**, *35*, 1–19, doi:10.1096/fj.202001792R.
38. Auwul, M.R.; Rahman, M.R.; Gov, E.; Shahjaman, M.; Moni, M.A. Bioinformatics and Machine Learning Approach Identifies Potential Drug Targets and Pathways in COVID-19. *Brief. Bioinform.* **2021**, *22*, doi:10.1093/BIB/BBAB120.
39. Mosharaf, M.P.; Reza, M.S.; Kibria, M.K.; Ahmed, F.F.; Kabir, M.H.; Hasan, S.; Mollah, M.N.H. Computational Identification of Host Genomic Biomarkers Highlighting Their Functions, Pathways and Regulators That Influence SARS-CoV-2 Infections and Drug Repurposing. *Sci. Reports 2022 121* **2022**, *12*, 1–22, doi:10.1038/s41598-022-08073-8.
40. Lee, H.; Park, J.; Im, H.J.; Na, K.J.; Choi, H. Discovery of Potential Imaging and Therapeutic Targets for Severe Inflammation in COVID-19 Patients. *Sci. Rep.* **2021**, *11*, 1–9, doi:10.1038/s41598-021-93743-2.

41. Alanazi, K.M.; Farah, M.A.; Hor, Y.Y. Multi-Targeted Approaches and Drug Repurposing Reveal Possible SARS-CoV-2 Inhibitors. *Vaccines* **2022**, *10*, doi:10.3390/vaccines10010024.
