# Supplementary material for: Meta-Data Analysis to Explore the Hub of the Hub-Genes That Influence SARS-CoV-2 Infections Highlighting Their Pathogenetic Processes and Drugs Repurposing
Source: Vaccines (Basel). 2022 Aug 3;10(8):1248. doi: 10.3390/vaccines10081248 (PMC9415433; doi:10.3390/vaccines10081248)
Supplement: Supplementary file 1 [file vaccines-10-01248-s001.zip › Supplementary table S2.pdf]

Supplementary Table S2a: 2D structures of the proposed drug agents/molecules.

S/N	Drug Name	2D Structure
1	Digoxin	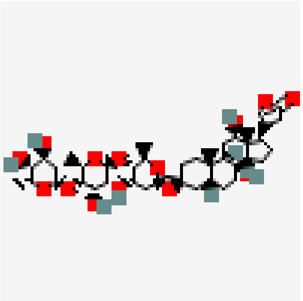
2	Avermectin	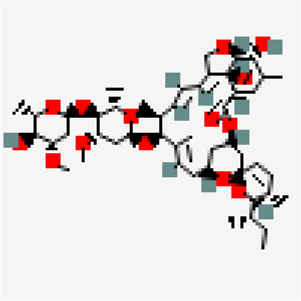
3	Simeprevir	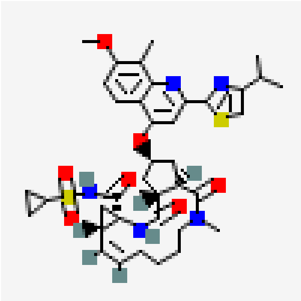
4	Linifanib	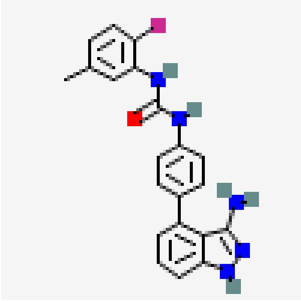
5	Nelfinavir Mesylate	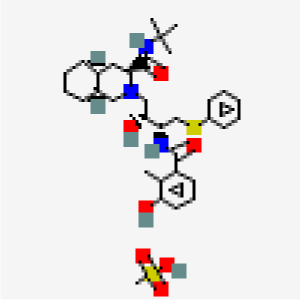

6	Atazanavir	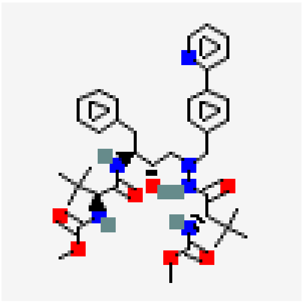
7	Withaferin	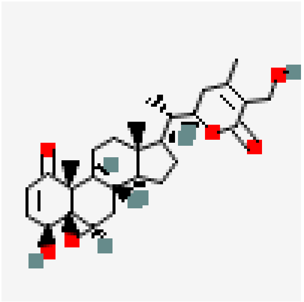
8	Proscillaridin	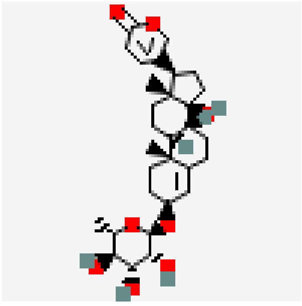
9	Hesperidin	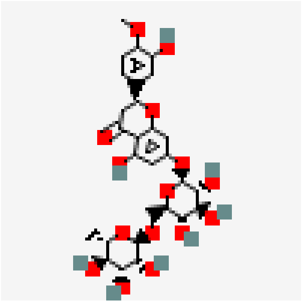
10	Amuvatinib	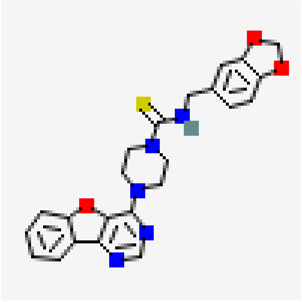

Supplementary Table S2b: Tanimoto's drug/chemical similarity coefficients

	Digoxin	Avermectin	Simeprevir	Linifanib	Nelfinavir Mesylate	Atazanavir	Withaferin	Proscillaridin	Hesperidin	Amuvatinib
**Digoxin**	1	0.65	0.34	0.23	0.29	0.29	0.51	0.53	0.38	0.28
**Avermectin**		1	0.49	0.29	0.36	0.38	0.64	0.53	0.45	0.39
**Simeprevir**			1	0.37	0.56	0.47	0.39	0.46	0.49	0.52
**Linifanib**				1	0.27	0.32	0.25	0.28	0.31	0.34
**Nelfinavir Mesylate**					1	0.42	0.32	0.35	0.38	0.39
**Atazanavir**						1	0.31	0.37	0.38	0.41
**Withaferin**							1	0.43	0.35	0.32
**Proscillaridin**								1	0.56	0.37
**Hesperidin**									1	0.42
**Amuvatinib**										1
